# Efficacy of Baduanjin Exercise for Sarcopenia in Older Adults: A 24‐Week Randomized Controlled Trial

**DOI:** 10.1002/jcsm.70163

**Published:** 2025-12-10

**Authors:** Keshangjing Wu, Xianyu Chen, Keyu Long, Shengnan Yue, Wenyuan Li, Xueqing Duan, Jing Zhang, Tianyu Liu, Yuanli Chen, Kaisy Xinhong Ye, Jing Guo, Bin Li

**Affiliations:** ^1^ Department of Geriatrics Hospital of Chengdu University of Traditional Chinese Medicine Chengdu China; ^2^ Yingmen Community Health Service Center of Jinniu District Chengdu China; ^3^ Evidence Based Traditional Chinese Medicine Center of Sichuan Province Hospital of Chengdu University of Traditional Chinese Medicine Chengdu China; ^4^ School of Sport and Health Chengdu University of Traditional Chinese Medicine Chengdu China; ^5^ Department of Psychological Medicine, Yong LooLin School of Medicine National University of Singapore Singapore Singapore; ^6^ Centre for Healthy Longevity, @AgeSingapore National University Health System Singapore Singapore; ^7^ Research Department & Teaching and Research Office of Epidemiology and Evidence‐Based Medicine Hospital of Chengdu University of Traditional Chinese Medicine Chengdu China

**Keywords:** Baduanjin, sarcopenia, short physical performance battery

## Abstract

**Background:**

Sarcopenia, a condition characterized by a substantial reduction in muscle mass and decreased muscle function, is a common degenerative condition in older adults. Regular Baduanjin exercise (BE) is deemed an effective intervention for improving muscle function in older adults. It is a traditional Chinese practice of *qigong* that combines gentle exercise, breathing techniques and meditation, in the elderly population. However, randomized controlled trials on the efficacy of BE in preventing and treating sarcopenia among older adults remain limited. The aim of our study was to assess the efficacy of a systematic BE regimen based on the short physical performance battery (SPPB) in managing sarcopenia. By establishing a clear link between BE and improvements in muscle function, this study reveals valuable insights into nonpharmacological strategies for the prevention and treatment of sarcopenia.

**Methods:**

This is a 24‐week randomized controlled trial involving 90 participants aged 60–77 years who were diagnosed with primary sarcopenia. The participants were randomly assigned by SAS software to either BE or a resistance training (RT) group. Both groups undertook their respective training under professional supervision from July 2022 to August 2023 in Chengdu, China. The intervention consisted of 60‐minute sessions, three times a week for 24 weeks. Participants were evaluated at baseline, Week 12 (midintervention) and Week 24 (postintervention) using the primary outcome measure, SPPB, along with secondary indicators, including limb muscle mass, two‐handed grip strength and other indicators, which were analysed using a generalized estimating equation (GEE) model.

**Results:**

Generalized estimating equations were used to assess 24‐week postintervention effects. Among the entire group, which consisted of 13 males and 77 females, significant differences (*p* < 0.05) were noted between the BE and RT in several measures: SPPB scores (*B* = 1.94 [95% CI, 1.20–2.68]), balance (*B* = 0.76 [95% CI, 0.31–1.22]), lower limb strength (*B* = 0.75 [95% CI, 0.25 to 1.24]), gait speed (*B* = 0.41 [95% CI, 0.01–0.81]), skeletal muscle index (SMI) (*B* = 0.37 [95% CI, 0.25–0.50]), left leg muscle mass (*B* = 0.38 [95% CI, 0.23–0.52]), right leg muscle mass (*B* = 0.34 [95% CI, 0.17–0.51]) and right hand grip strength (*B* = 1.56 [95% CI, 1.13–1.99]). No significant differences were found in the muscle mass of the left arm, right arm or left handgrip strength or in the results of the 6‐m walk test.

**Conclusions:**

Regular BE markedly improved muscle function and increased muscle mass in older adults with sarcopenia, indicating a safe and effective exercise for sarcopenia management. BE, which was tailored using the short physical performance battery assessment, offers a valuable approach to help prevent and treat sarcopenia in older adults. Future research should investigate the long‐term effects of varying BE intensities and frequencies in individuals with sarcopenia.

**Trial Registration:** Chinese Clinical Trial Registry (ChiCTR2100051871).

**Abbreviations**
BMI: body mass index
GEE: generalized estimating equation
ICD: International Classification of Diseases
ITT: intent‐to‐treat
PPS: per‐protocol set
RCTs: randomized clinical trials
SMI: skeletal muscle index
SPPB: short physical performance battery
TCM: traditional Chinese medicine

## 1 Introduction

The global population is aging at an unprecedented rate, making aging a critical issue. Sarcopenia, a muscle‐wasting disease that primarily affects older adults, has become a significant health concern in this demographic shift. This condition is marked by a progressive loss of muscle mass and strength, which can greatly impair quality of life and independence. Epidemiological data show that the prevalence of sarcopenia among elderly individuals in China is approximately 9.8%–12.0% in women and 6.7% in men, with higher rates in rural areas (13.1%), compared to urban areas (7.0%) [1]. Projections further indicate that by 2045, the number of individuals with sarcopenia in Europe will increase by 72.4%, rising from 10 869 527 in 2016 to 18 735 173, whereas prevalence among the elderly will grow from 11.1% to 12.9% [2]. Additionally, studies demonstrate a strong association between sarcopenia and other conditions such as diabetes, with prevalence reaching 15.9% among diabetic older adults compared to 10.8% in nondiabetic ones [3]. Collectively, these findings underscore that sarcopenia is a pressing global public health challenge.

As a chronic condition that accelerates aging, sarcopenia is influenced by complex and multifactorial pathogenic factors. It can be categorized as either primary or secondary. Primary sarcopenia is age‐related and occurs in the absence of identifiable causes, whereas secondary sarcopenia arises from factors such as physical inactivity, chronic diseases (e.g., heart failure and cancer) or inadequate nutrition. Primary sarcopenia, a natural consequence of aging, is characterized by gradual declines in muscle mass and function without underlying disease [4]. Both domestic and international studies indicate that elderly individuals with low physical activity levels are particularly prone to developing primary sarcopenia [5]. Current therapeutic strategies include exercise interventions, nutritional support and pharmacotherapy [6, 7]. Although no medications have yet been approved specifically for sarcopenia, treatments such as testosterone supplementations are under investigation. These therapies, especially when combined with exercise and nutrition, may improve muscle mass and strength [8]. Resistance training, in particular, is recognized as one of the most effective interventions, significantly enhancing muscle mass and strength in older adults.

Despite its benefits, RT can be difficult for many elderly individuals because of age‐related limitations, comorbidities or restricted living conditions. Therefore, identifying exercise methods that are safe, accessible and effective is crucial. In recent years, traditional Chinese medicine (TCM) exercises such as Baduanjin have gained recognition for their health benefits in the elderly. Baduanjin, a widely practiced form of TCM exercise, is easy to learn, safe and effective. Long‐term practice has been shown to improve balance, posture and disease management. Research has indicated that TCM exercises, such as Baduanjin, provide significant benefits for elderly patients with sarcopenia [9]. Other traditional *qigong* practices and herbal therapies have also been shown to enhance physical performance and muscle strength in older adults [10]. Baduanjin, as a form of *qigong*, has additional benefits, including stress reduction, respiratory muscle strengthening and immune enhancement, which may aid in preventing respiratory infections [11]. Mild adverse effects such as muscle soreness, fatigue and dizziness have been reported but remain rare and not severe [12]. Furthermore, combining exercise with nutritional supplementation has demonstrated improvements in muscle strength and physical function, such as sit‐to‐stand performance, among community‐dwelling elderly Chinese patients with sarcopenia [13]. Baduanjin has also been shown to increase muscular strength in sarcopenia patients undergoing maintenance haemodialysis [14]. However, randomized controlled trials (RCTs) specifically evaluating Baduanjin for primary sarcopenia in older adults, particularly with tools such as the SPPB, are still lacking.

Many existing studies emphasize physiological markers (e.g., muscle strength and balance) while overlooking functional outcomes and quality of life, which are equally critical for assessing intervention effectiveness in elderly populations. The short physical performance battery (SPPB) is a key tool for evaluating lower extremity function in older adults. It comprises a balance test, a sit‐to‐stand test and a gait speed test, collectively offering a comprehensive assessment of physical function. Beyond diagnosis, the SPPB has predictive value, identifying risks such as falls, functional decline and even mortality. Importantly, reference values vary across populations; for instance, those established in Norway [15] differ from those in Singapore [16], where the SPPB has been applied to evaluate somatic manifestations of sarcopenia. To evaluate differences in the effects of regular BE and RT in older adults with primary sarcopenia, this study employed the SPPB scale to compare outcomes between the two interventions. Evidence supporting nonpharmacological approaches to sarcopenia is steadily growing. In this context, BE emerges as a safe, low‐impact and cost‐effective option for older adults.

## 2 Methods

### 2.1 Design Overview

This study was conducted from July 2022 to August 2023 at the Chengdu University of Traditional Chinese Medicine Hospital and its surrounding community. Eligible participants were initially screened using questionnaires, followed by discussions, public awareness activities and educational sessions. Informed consent was subsequently obtained, and consent forms were signed. The detailed study protocol and statistical analysis plan, previously published [17] are described in the methodology section and available in Supporting Information S1.

A total of 90 older adults diagnosed with primary sarcopenia (ICD code is M62.84) were enrolled by a licensed geriatrician with more than 10 years of clinical experience. Participants were randomly assigned to either the BE group or the RT group. After 24 weeks of intervention, both groups were assessed to evaluate the clinical effectiveness of the standard BE program in elderly individuals with sarcopenia. The primary aim was to investigate the potential of BE as an effective intervention for sarcopenia and to inform future prevention and treatment strategies.

The study protocol received ethical approval from all participating institutions, complied with the principles of the Declaration of Helsinki and was registered with the Chinese Clinical Trial Registry (ChiCTR2100051871) under ethical review approval number 2021KL‐058. The research adhered to the Consolidated Standards of Reporting Trials (CONSORT) guidelines.

### 2.2 Underlying Conceptual Structure of the Research

**FIGURE 1:**
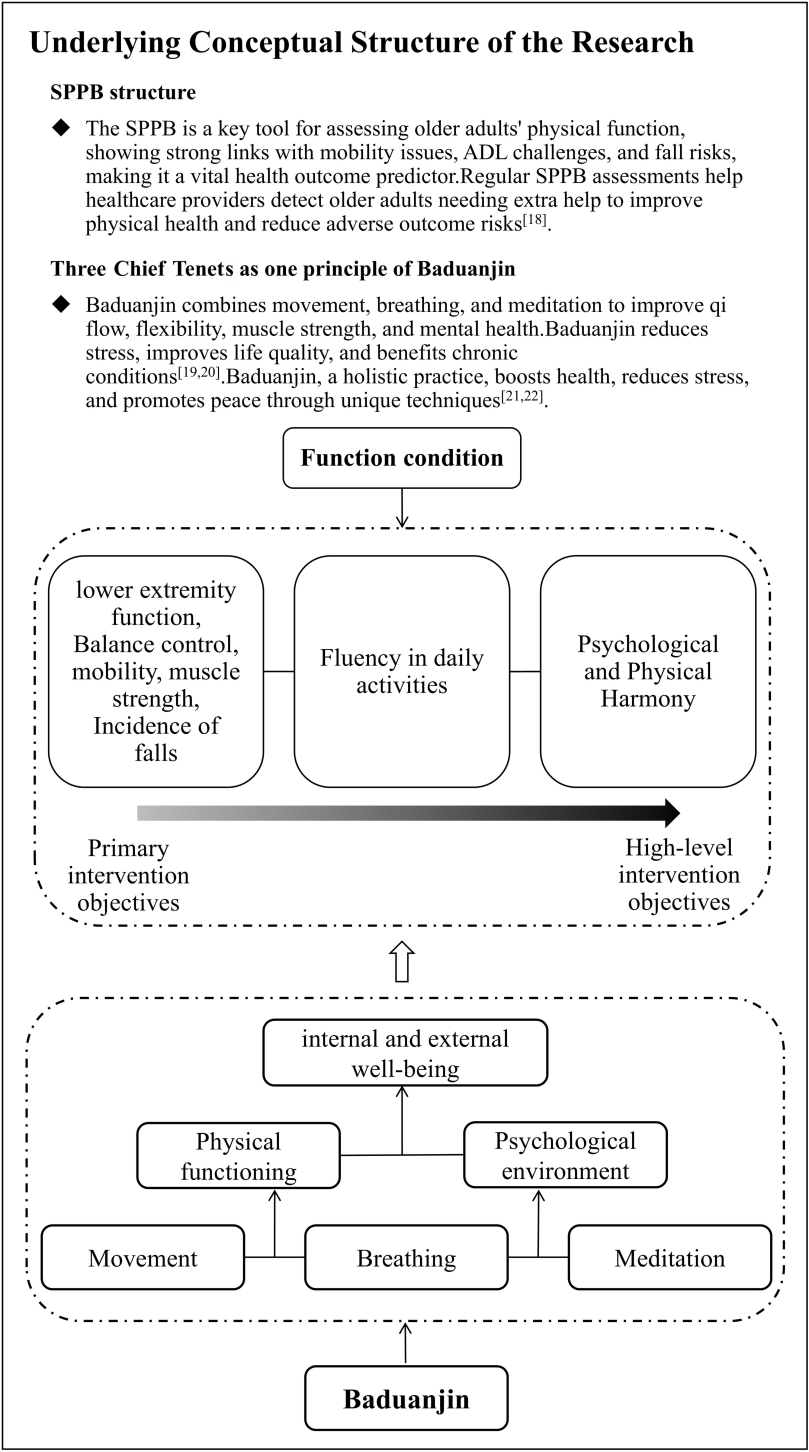
Underlying conceptual structure of the research. Conceptual structure: the combination of three chief tenets as one theory of Baduanjin and the SPPB structure. SPPB indicates short physical performance battery.

This study employed both the SPPB framework and the three chief tenets as one theory of Baduanjin (Figure 1).

### 2.3 Participants

Participants were recruited from the Affiliated Hospital of Chengdu University of TCM and the surrounding neighbourhoods. Advertisements, questionnaires and posters were used as modes of recruitment. During the recruitment, initial screening procedures included measurements of calf circumference, two‐handed grip strength and five‐time sit‐up duration (gait speed). Individuals suspected of having sarcopenia underwent body composition analysis. Based on sample size estimation principles [18] and previous trials [19], each group required 45 patients to achieve 90% statistical power with a 5% significance level. Ultimately, 90 eligible older adults with sarcopenia were enrolled from an initial pool of 123 candidates, following the diagnostic criteria established by the Asian Working Group for Sarcopenia (AWGS) [20].

According to AWGS, sarcopenia is defined as low skeletal muscle mass (SMI ≤ 7.0 kg/m^2^ for men and ≤ 5.7 kg/m^2^ for women), measured by bioelectrical impedance analysis (BIA) in combination with either low muscle strength (handgrip < 28 kg for men; <18 kg for women) or poor physical performance (6‐m walk < 1.0 m/s, five‐time chair stand ≥ 12 s or SPPB ≤ 9).

Eligibility criteria included participants aged 60–84 years who were clinically diagnosed with sarcopenia and able to complete body composition analysis, grip strength assessment, 6‐m walk tests and questionnaires. Participants were also required to walk (with or without support devices), safely perform prescribed exercises under professional supervision and provide written informed consent.

Exclusion criteria included walking speed exceeding 1.2 m/s, a five‐time sit‐to‐stand duration ≤ 12 s or an SPPB score > 9. Individuals with severe conditions affecting muscle or bone metabolism, such as advanced renal disease, malignancy, cerebrovascular events, severe liver disease, uncontrolled diabetes or thyroid and parathyroid disorders, were excluded. Participants taking medications that influence muscle or bone metabolism, those engaged in recent regular high‐intensity exercise or plyometric training, and individuals with significant cognitive impairment (Brief Intelligence Scale score < 20) were also excluded. Comprehensive inclusion, exclusion and loss‐to‐follow‐up criteria are provided in Table S1.

### 2.4 Randomization and Blinding

Upon enrolment, the study participants were randomly assigned to either the BE group or the RT group, which was also the control group, at a 1:1 ratio. The randomization sequence was secured in a sealed envelope by independent reviewers, who were blinded to the study. After completing all baseline assessments, participants were informed of their group assignment via telephone. Given the nature of the behavioural intervention, the participants, athletic trainers and intervention supervisors were aware of the group assignments. However, outcome assessors and statistical analysts remained blinded to these assignments, with group identifiers replaced by letters A and B on the case report forms. The blinding was maintained until all the statistical analyses were fully completed.

### 2.5 Interventions

Participants in both groups received 10 min of health education weekly, focusing on nutrition and exercise for sarcopenia management [21, 22]. The BE group engaged in structured training sessions conducted by a senior BE coach, according to the 2018 guidelines established by the State General Administration of Sports [23]. All sessions were conducted at a consistent time and location following the initial health education program. Similarly, participants in the RT group attended sessions led by a qualified kinesiology professor. These sessions were also scheduled at fixed times and locations, determined by the geographical distribution of the research subcentres. To ensure adherence to treatment protocols, quality control staff conducted weekly telephone follow‐ups for both groups.

**FIGURE 2:**
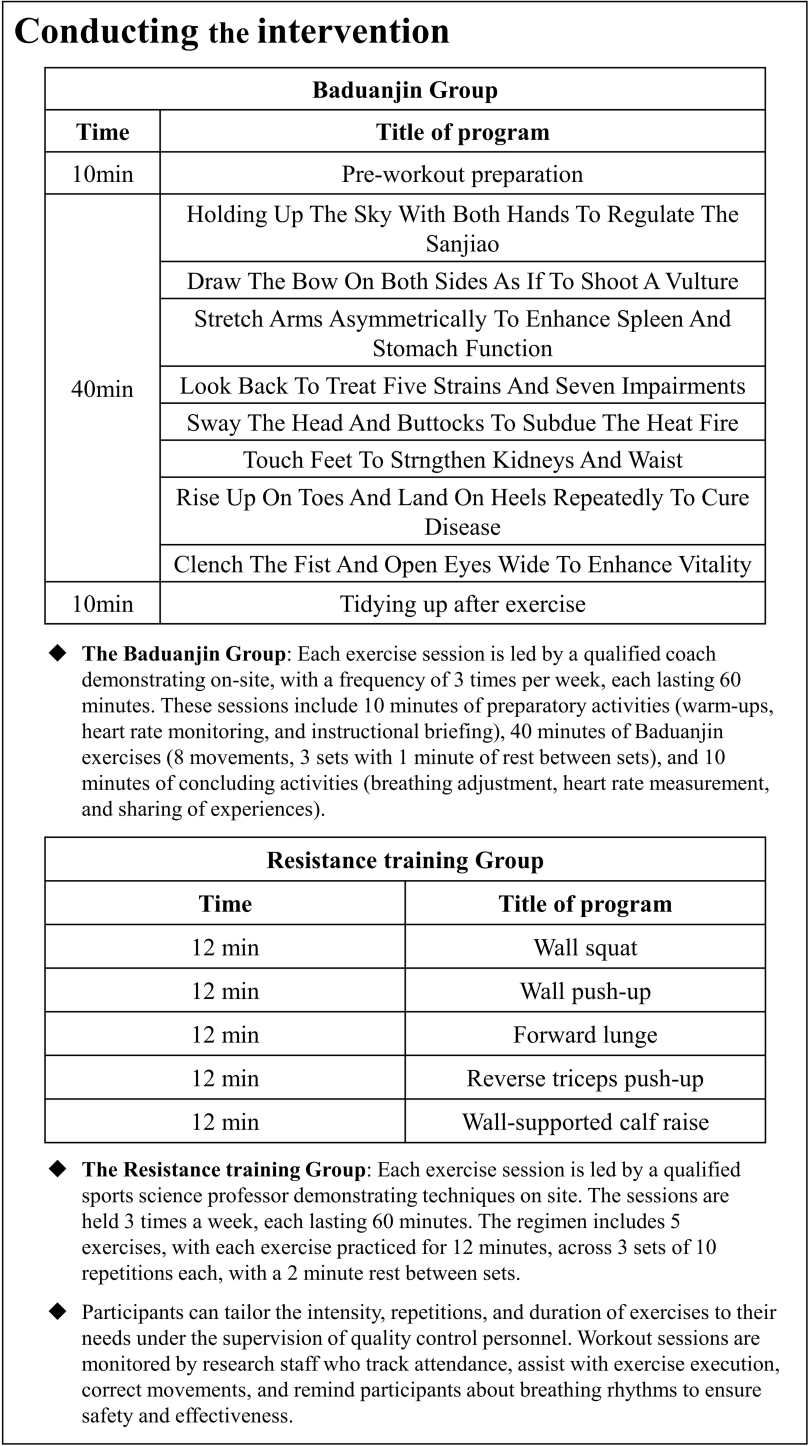
Baduanjin program intervention components.

**FIGURE 3:**
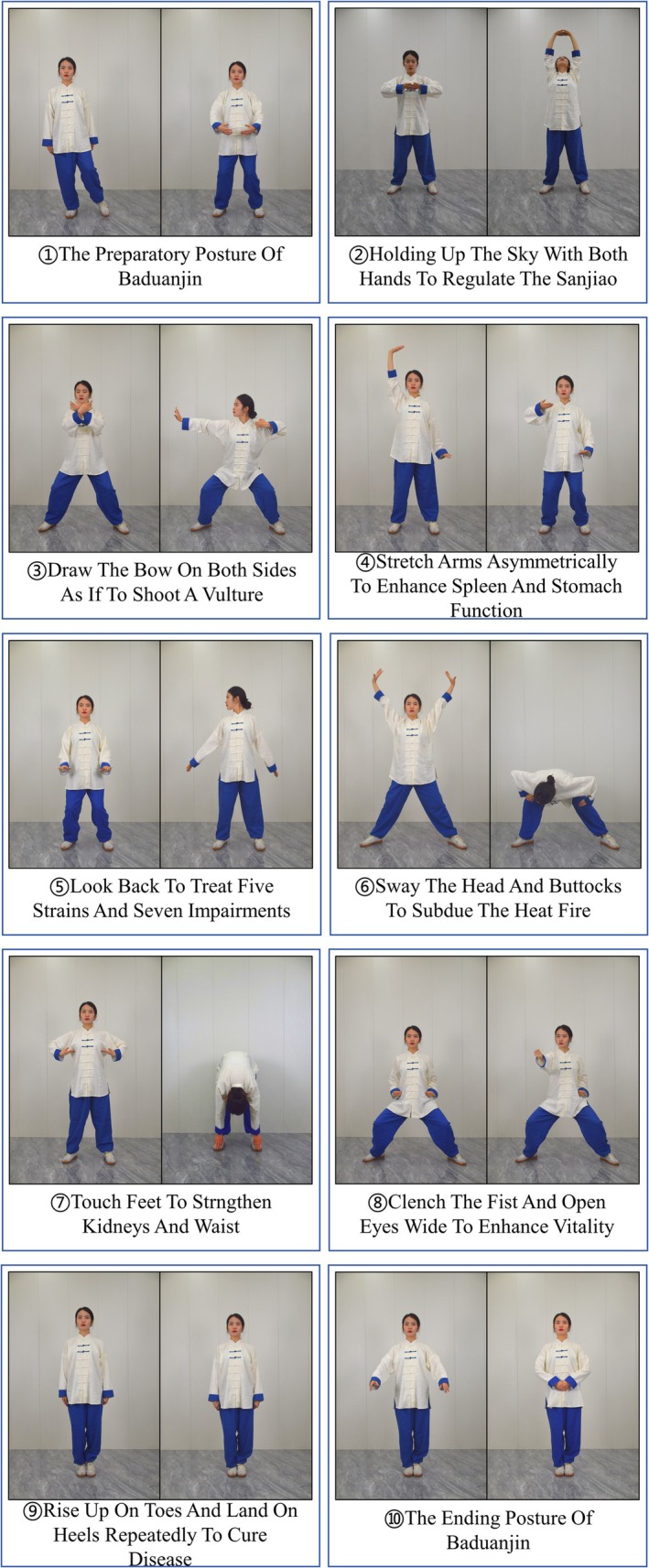
Examples of movements of each action.

The training protocols and a detailed description of each group's activities are presented in Figure 2. A breakdown of the BE movements is illustrated in Figure 3, and demonstrations of the RT exercises are provided in Figures S1–S5 (Supporting Information S2). Throughout the study period, all participants were required to record their daily activity levels, any symptoms of discomfort and maximum heart rates experienced during training.

### 2.6 Assessments and Outcomes

The primary outcome measure for this study was the SPPB score, which was assessed at baseline, Week 12 and Week 24. The SPPB, endorsed by the US National Institute on Aging, is a standardized tool used to evaluate muscle function in older adults. It comprises three components: a balance test, the repeated five‐time sit‐to‐stand test and a gait speed test [24]. Based on prior research demonstrating clinically meaningful improvements in the functional mobility of older adults, a three‐point change has been identified as the minimal clinically important difference (MCID) [25]. Each component is scored on a scale of 0–4, yielding a total maximum score of 12, with higher scores indicating better physical performance. For accuracy, each test component was repeated two to three times, and the shortest completion time was recorded. Detailed SPPB scoring criteria are provided in Table S2.

This study assessed both primary outcomes, including limb mobility and SMI improvement, and secondary outcomes, such as muscle mass, grip strength, 6‐m walking time and the incidence of falls. Muscle mass was measured using a body composition analyser (Donghuayuan DBA‐510, Beijing, China; Figure S26), which provided detailed body composition metrics including muscle mass, body weight and body mass index (BMI). Measurements were taken at three key time points: baseline (Week 1), midintervention (Week 12) and postintervention (Week 24). Data from the analyser were used to calculate the SMI across different body regions, including the torso, upper limbs and lower limbs [26].

Grip strength, an indicator of upper limb strength, was evaluated at the same intervals (baseline, Week 12 and Week 24) using a JAMAR grip strength meter (CAMRY EH101, Yiwu, China; Figure S27), with higher readings reflecting greater strength. The 6‐m walk test measured the time required to walk a straight 6‐m distance, and gait speed was recorded to two decimal places.

The incidence of falls was monitored throughout the study at Weeks 1, 4, 8, 12, 16, 20 and 24. All fall events and any subsequent medical interventions were documented. A fall was defined as an unintentional descent to the ground, floor or lower level, occurring on flat surfaces, stairs or when colliding with objects such as furniture.

### 2.7 Statistical Analysis

Under the intent‐to‐treat (ITT) framework, participants who completed between 80% and 120% of the prescribed exercise regimen were included in the analysis. Categorical variables were expressed as frequencies and percentages to describe the distribution of discrete characteristics, such as sex and health condition. Continuous variables were summarized as means ± standard deviations for normally distributed data and as medians with interquartile ranges for nonnormally distributed data, providing robust measures of central tendency and dispersion.

Comparison between groups was conducted using *t*‐tests for continuous variables and chi‐square tests or Fisher's exact tests for categorical variables, depending on the sample size. SPSS version 27.0 and GraphPad Prism version 7.0 were used for the statistical analysis.

Given the repeated measurements of outcome indicators, a generalized estimating equation (GEE) model was applied to analyse longitudinal data over a 24‐week study period. In this model, outcome differences from baseline were treated as dependent variables, whereas group assignment served as the independent variable. Missing data were handled directly within the GEE framework. Sex was included as a covariate to control for potential confounding effects.

An interaction term was included in the regression model to assess whether the mean differences between groups changed significantly over time. Specifically, the model included time (categorical: baseline, Week 12 and Week 24), group (intervention vs. control) and the time × group interaction, allowing for evaluation of differential temporal effects between groups. Statistical significance was set at a *p* < 0.05 (two‐sided). No imputation or additional data handling methods were applied in the primary analysis.

## 3 Results

### 3.1 Participants' Characteristics

**FIGURE 4:**
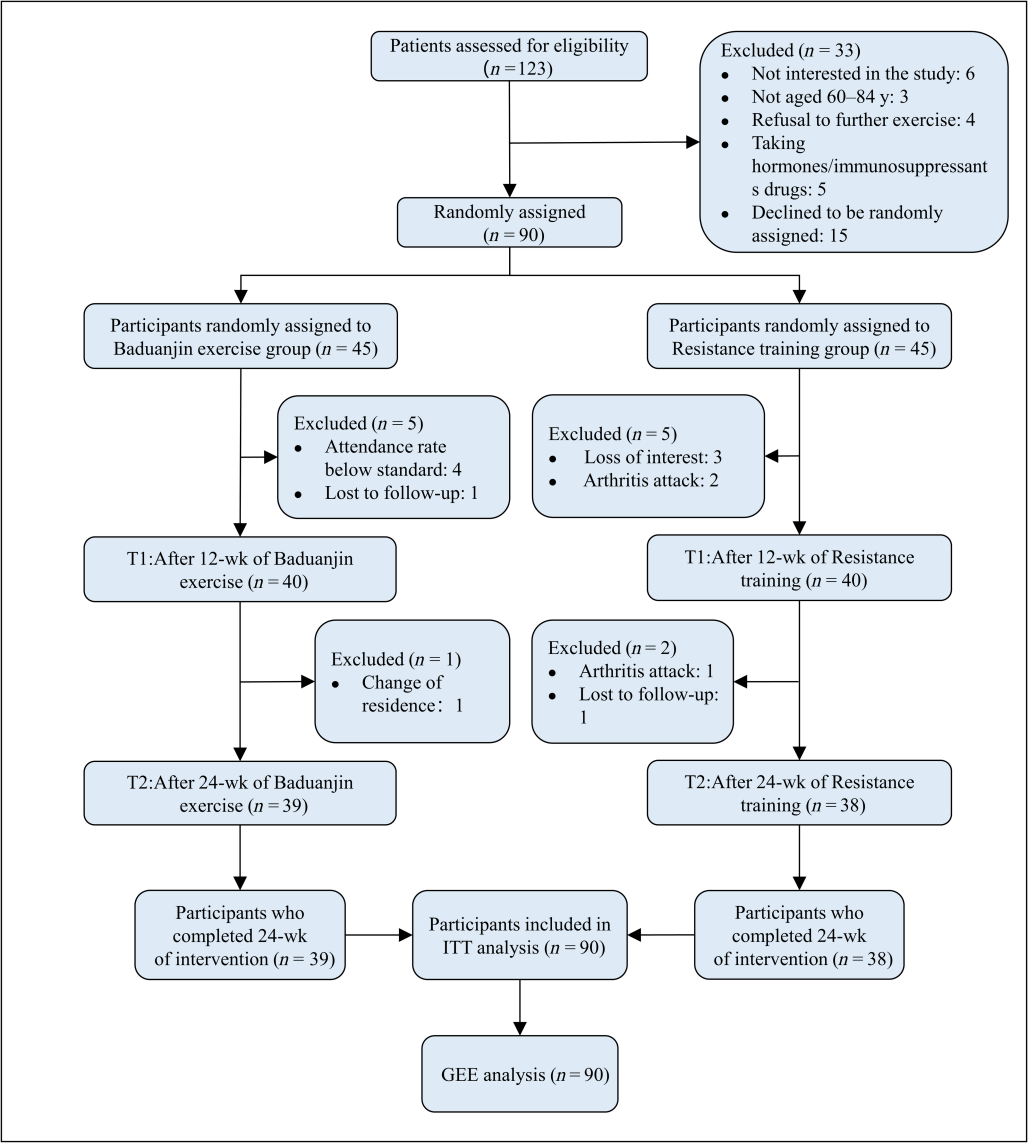
Flow diagram of participant enrolment.

A total of 90 participants met the inclusion criteria at the initial screening. However, 13 were lost to follow‐up leaving 77 for the final study (Figure 4 shows the selection process). The average age of participants was 67.31 years (standard deviation, SD = 5.57); the average height was 1.60 m (SD = 0.67), and the majority were female. Most participants were nonreligious, well educated, married, financially stable and not engaged in regular physical exercise. Typically, subjects had no more than two chronic diseases. The majority could not adhere to their regular medication regimens. A small fraction of the cohort exhibited mild cognitive impairment. No significant differences were observed in the sociodemographic indicators between the two groups, except for height (*t* = −3.634; *p* < 0.001). Table 1 presents the baseline analysis of the sociodemographic profiles. Data for Weeks 12 and 24 can be found in Supporting Information S2.

**TABLE 1:** Baseline characteristics of the participants.

Characteristics	All (*n* = 90)	Baduanjin Group (*n* = 45)	Resistance training group (*n* = 45)	χ ^2^	*p*
	*N*(%)/SD	*N*(%)/SD	*N*(%)/SD		
Gender				0.09	0.764
Male	13(14.44%)	6(13.33%)	7(15.56%)		
Female	77(85.56%)	39(86.67%)	38(84.44%)		
Marital status				1.353	0.245
Married	76(84.44%)	40(88.89%)	36(80.00%)		
Single/widowed/divorce/separated	14(15.56%)	5(11.11%)	9(20.00%)		
Educational level				0.485	0.784
Primary school or less	29(32.22%)	15(33.33%)	14(31.11%)		
Secondary school	34(37.77%)	18(40%)	16(35.56%)		
College and above	27(30.00%)	12(26.67%)	15(33.33%)		
Cognitive level				1.111	0.292
Normal cognitive level	81(90.00%)	42(93.33%)	39(86.67%)		
Mild cognitive impairment	9(10.00%)	3(6.67%)	6(13.33%)		
Regular exercise quantity				0.407	0.523
Never	51(56.67%)	24(53.33%)	27(60.00%)		
Occasionally	39(43.33%)	21(46.67%)	18(40.00%)		
Chronic diseases				0.452	0.798
None	33(36.67%)	17(37.78%)	16(35.56%)		
One to two types of diseases	47(52.22%)	24(53.33%)	23(51.11%)		
More than two diseases	10(11.11%)	4(8.89%)	6(13.33%)		
Religion				0	1.000
None	86(95.56%)	43(95.56%)	43(95.56%)		
Others	4(04.44%)	2(04.44%)	2(04.44%)		
Medication adherence status				5.553	0.135
None	64(71.11%)	33(73.33%)	31(68.89%)		
One type of drug	11(12.22%)	6(13.33%)	5(11.11%)		
Two types of drug	10(11.11%)	6(13.33%)	4(08.89%)		
More than two drugs	5(05.56%)	0(00.00%)	5(11.11%)		
Economic condition				1.668	0.197
Enough	71(78.89%)	38(84.44%)	33(73.33%)		
Not enough	19(21.11%)	7(15.56%)	12(26.67%)		
	Mean ± SD			*t*	*p*
Age	67.31 ± 5.57	66.27 ± 5.54	68.36 ± 5.45	−1.803	0.075^a^
Height	1.60 ± 0.67	1.57 ± 0.71	1.62 ± 0.53	−3.634	0.001^a^

^a^ *p* < 0.05.

### 3.2 Differences Between Groups in the Impact of BE on SPPB Scores Over Time

**FIGURE 5:**
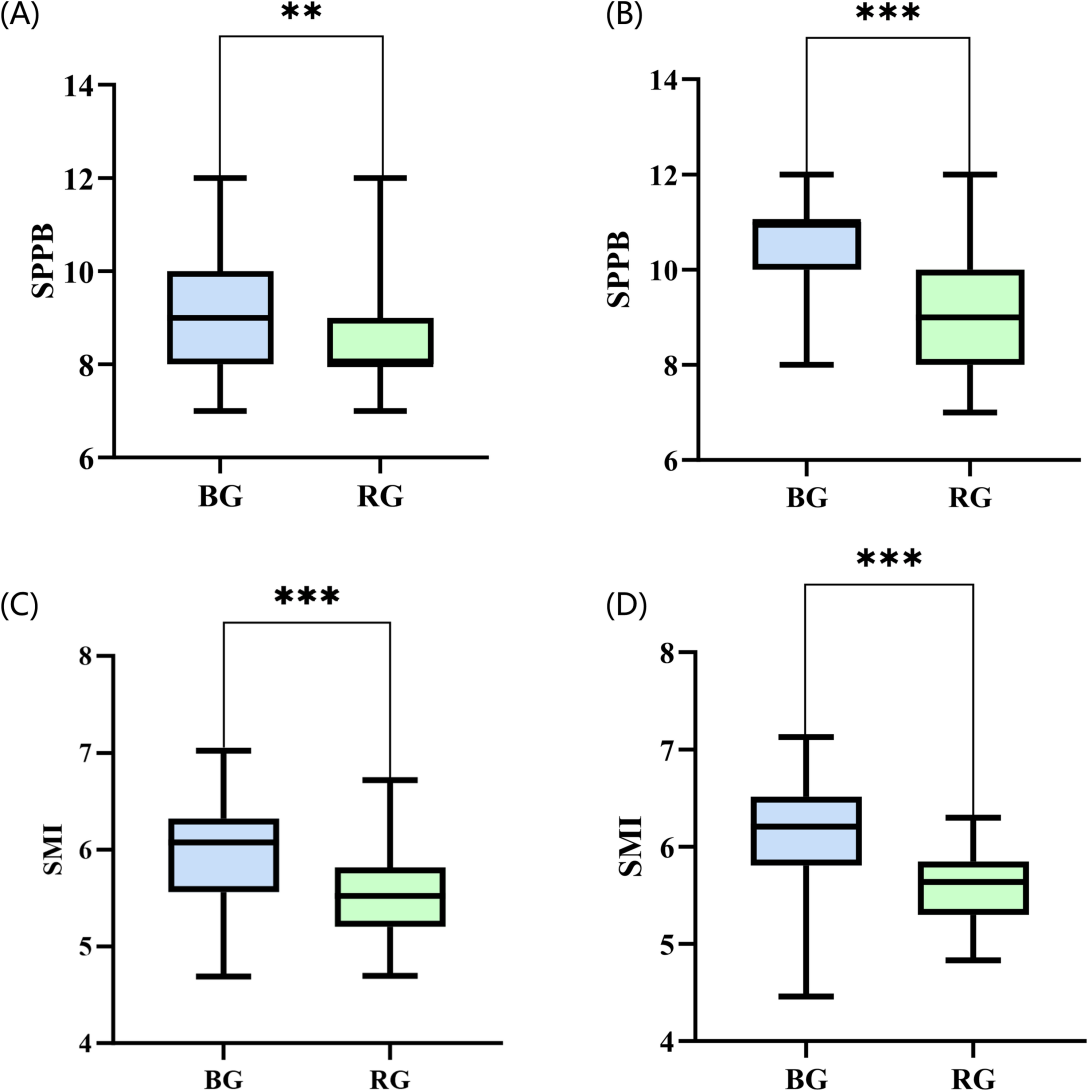
Changes in SPPB and SMI. (A) Comparison of SPPB scores between the BE group and the RT group at the Week 12 time point. (B) Comparison of SPPB scores between the BE group and the RT group at the Week 24 time point. (C) Comparison of the SMI between the BE group and the RT group at the Week 12 time point. (D) Comparison of the SMI between the BE group and the RT group at the Week 24 time point. In the box plots, black dots represent the means, horizontal lines represent the medians, and the top and bottom edges of the boxes represent the upper and lower quartiles, respectively. Bottom: In the line plot, all the means and their corresponding 95% confidence intervals (CIs) were estimated using a linear regression model.

Table 2 shows the outcomes from the GEE analysis comparing the BE and RT groups, with adjustments for baseline values. Significant differences were observed at both the 12‐week (T1) and 24‐week (T2) assessments postintervention. At T1, BE presented significantly better outcomes than the RT did in terms of SPPB (*B* = 0.97 [95% CI, 0.28 to 1.65]) (Figure 5A) and gait speed (*B* = 0.28 [95% CI, 0.04 to 0.52]). No significant differences were found in lower limb muscular strength (*B* = 0.41 [95% CI, −0.04 to 0.85]) or balance (*B* = 0.29 [95% CI, −0.09 to 0.66]). At T2, notable improvements were again observed in BE, with significant increases in SPPB (*B* = 1.94 [95% CI, 1.20–2.68]) (Figure 5B), balance (*B* = 0.76 [95% CI, 0.31–1.22]), lower limb muscular ability (*B* = 0.75 [95% CI, 0.25–1.24]) and gait speed (*B* = 0.41 [95% CI, 0.01–0.81]).

**TABLE 2:** Changes for primary and secondary outcome variables across time.

Variable	Range^a^	Desired direction^b^	Generalized estimating equation models					
			T0^c^		T1^d^		T2^e^	
			95% CI	*p*	95% CI	*p*	95% CI	*p*
Short physical performance battery (SPPB)	0–12	↑	−0.20 (−0.52, 0.12)	0.228	0.97 (0.28, 1.65)	0.006^f^	1.94 (1.20, 2.68)	0.001^f^
Balance	0–4	↑	−0.03 (−0.25, 0.19)	0.822	0.29 (−0.09, 0.66)	0.135	0.76 (0.31, 1.22)	0.001^f^
Lower limb strength	0–4	↑	−0.26 (−0.51, −0.01)	0.040^f^	0.41 (−0.04, 0.85)	0.073	0.75 (0.25, 1.24)	0.003^f^
Gait speed	0–4	↑	0.02 (−0.12, 0.16)	0.811	0.28 (0.04, 0.52)	0.023^f^	0.41 (0.01, 0.81)	0.046
Skeletal muscle index (SMI) (kg/m^2^)	\	↑	‐0.01 (−0.04, 0.01)	0.318	0.31 (0.20, 0.42)	0.001^f^	0.37 (0.25, 0.50)	0.001^f^
Arm muscle mass (left) (kg)	\	↑	0.00 (−0.02, 0.02)	0.841	0.08 (−0.02, 0.17)	0.109	0.00 (−0.05, 0.05)	0.904
Arm muscle mass (right) (kg)	\	↑	0.01 (−0.01, 0.03)	0.388	0.01 (−0.03, 0.06)	0.545	0.02 (−0.04, 0.08)	0.527
Leg muscle mass (left) (kg)	\	↑	−0.00 (−0.04, 0.03)	0.815	0.26 (0.13, 0.40)	0.001^f^	0.38 (0.23, 0.52)	0.001^f^
Leg muscle mass (right) (kg)	\	↑	−0.01 (−0.05, 0.03)	0.546	0.24 (0.09, 0.38)	0.001	0.34 (0.17, 0.51)	0.001^f^
Handgrip strength								
Hand (left) (kg)	\	↑	−0.37 (−0.81, 0.07)	0.102	0.51 (−0.33, 1.35)	0.233	0.86 (−0.11, 1.82)	0.084
Hand (right) (kg)	\	↑	−0.05 (−0.14, 0.04)	0.294	1.20 (0.89, 1.51)	0.001^f^	1.56 (1.13, 1.99)	0.001^f^
6‐m walk test (m/s)	\	↓	−0.00 (−0.02, 0.02)	0.658	−0.00 (−0.06, 0.06)	0.987	−0.01 (−0.09,0.07)	0.798

^a^ The range delineates the normal value boundaries for the primary observed metric, establishing its minimum and maximum thresholds. As for the secondary outcomes, because of the absence of a standardized measure, it is represented by the symbol ‘\’.

^b^ The desired direction reflects the behaviour of the outcomes being monitored. An upward‐pointing arrow signifies that an increase in the outcomes correlates with an improvement in conditions, whereas a downward‐pointing arrow indicates that a decrease in the outcomes is associated with better outcomes.

^c^ T0: the 0th week.

^d^ T1: the 12th week.

^e^ T2: the 24th week.

^f^ *p* < 0.05.

### 3.3 Differences Between Groups in the Impact of BE on Secondary Outcome Scores Over Time

**FIGURE 6:**
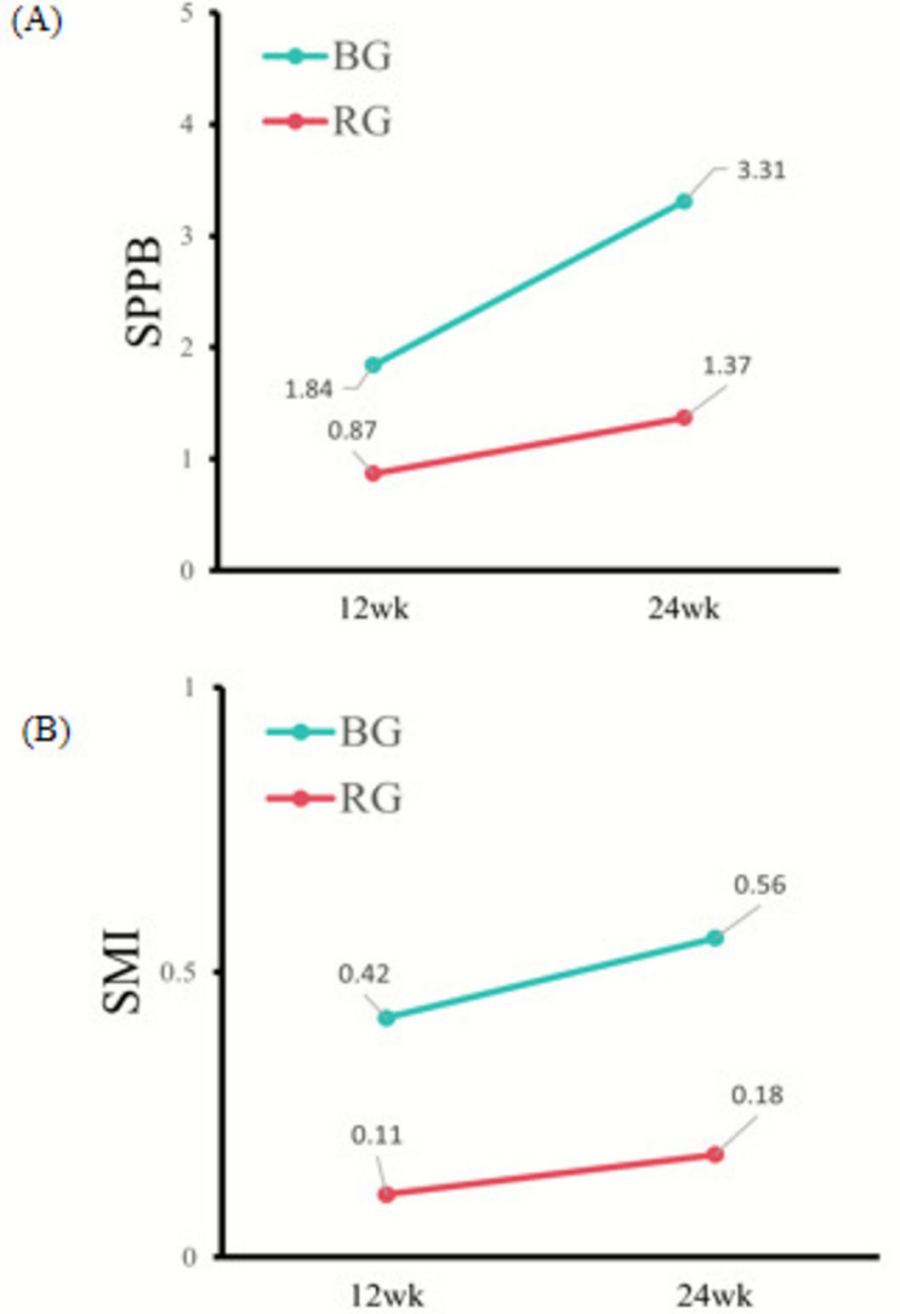
Changes in SPPB and SMI. (A) Within‐group differences in SPPB scores between the BE group and the RT group. (B) Within‐group differences in SMI scores between the BE group and the RT group.

At the Week 12 intervention, there was a significant between‐group difference in SMI. Specifically, the SMI (*B* = 0.31 [95% CI, 0.20–0.42]), left leg muscle mass (*B* = 0.26 [95% CI, 0.13–0.40]), right leg muscle mass (*B* = 0.24 [95% CI, 0.09–0.38]) and right hand grip strength (*B* = 1.20 [95% CI, 0.89–1.51]) were greater in the BEgroup than in the RT group (Figure 5C). After 24 weeks of intervention, compared with the RT group, the BE group demonstrated significant improvements in the SMI (*B* = 0.37 [95% CI, 0.25–0.50]) (Figure 5D), left leg muscle mass (*B* = 0.38 [95% CI, 0.23–0.52]), right leg muscle mass (*B* = 0.34 [95% CI, 0.17–0.51]) and right hand grip strength (*B* = 1.56 [95% CI, 1.13–1.99]). In contrast, bilateral upper extremity muscle mass, left hand grip strength and the results of the 6‐m walk test did not significantly differ between the intervention and control groups at Week 12 or 24. The within‐group differences are shown in Figure 6. The changes in the median of each observed indicator are shown in Figures S6–S25.

### 3.4 Intervention Adherence and Adverse Events

The study reported an overall dropout rate of 14.4% (BE: 13.3%; RT: 15.6%). Participants who engaged in at least 80% of the total prescribed sessions were considered to have met the study's inclusion criteria for analysis. Among the 77 included participants, the adherence rate was 100%. There were 24 participants (53.3%) from the BE group who indicated their intention to continue BE in the future. Falls did not occur in either group. No adverse events were reported throughout the duration of the study.

## 4 Discussion

This RCT employed the SPPB as the primary benchmark for evaluating the intervention's effectiveness. The BE program led to a significant improvement in functional body control and increased muscle mass in the lower extremities at both the midintervention (T1) and the postintervention (T2) assessments. In addition, participants in the intervention group demonstrated a marked enhancement in right‐hand grip strength. Furthermore, several participants reported alleviation of symptoms associated with chronic conditions following their engagement in the exercise regimen.

According to our experimental findings, the primary indicator of balance showed a significant improvement at Week 24 compared with Week 12. In contrast, lower limb strength did not demonstrate a significant increase at Week 12 but improved markedly by Week 24. This trend likely reflects the fact that measurable gains in muscle strength typically require a longer duration of systematic training to reach statistical significance [27, 28]. The observed enhancement in physical function can be attributed to the emphasis on balance and flexibility inherent in BE. These movements challenge proprioceptive control and enhance neuromuscular coordination key factors for maintaining stability in older adults.

All outcomes were assessed using the SPPB, a validated tool for evaluating lower limb function in older populations, highlighting the importance of long‐term, structured interventions to improve overall physical performance. Notably, all three test components of the SPPB demonstrated maximal improvement by Week 24, indicating substantial gains in lower limb function. These findings align with previous studies reporting the benefit of *qigong*‐based interventions on physical function among older adults [29]. BE promotes increased joint range of motion and muscle endurance, thereby enhancing performance in tasks that require coordination and lower limb strength.

Among the secondary outcomes, BE was more effective in promoting muscle growth in the lower limbs than in the upper limbs. This difference is likely due to the targeted activation of lower limb muscles in many Baduanjin movements, which require lower body support and balance, consequently improving strength, coordination and postural stability [30, 31]. Grip strength—a key indicator of muscle function and overall muscle mass—also significantly improved in participants following the BE program. This improvement may be attributed to the coordinated engagement of upper limb and core muscles alongside lower limb movements, thereby enhancing overall muscle performance [32].

Although left‐handed grip strength did not improve significantly, some variability was observed; this result may be due to the limited sample size. Further research is warranted to explore this aspect in greater depth. The change in walking speed between the two time points was not significant, possibly due to multiple contributing factors affecting gait speed or an early plateau effect in the intervention group [33].

RT requires the use of specialized equipment and a certain level of training intensity; therefore, exercise plans must be individualized according to each participant's chronic disease status. In addition, continuous supervision is necessary during training to ensure safety and prevent accidents, which can inadvertently hinder the widespread implementation of RT among older adults.

In contrast, BE, a traditional Chinese health exercise, uniquely integrates physical movement, breath control and mental relaxation. Characterized by its moderate intensity, BE combines physical and mental practices within the framework of TCM meridian theory, allowing participants to directly experience the principle of TCM. This integration enhances both engagement and adherence.

The holistic nature of BE is likely to exert comprehensive benefits on muscle function and overall health in older adults. By alleviating muscle fibre pain—commonly experienced in this population—BE helps improve balance and stability [34]. These benefits are partly attributed to its targeted stimulation of lower limb function. Moreover, regular BE practice may help slow the progression of various chronic conditions, including psychological disorders, by promoting both physical and mental well‐being [35, 36].

Although numerous clinical studies and meta‐analyses have examined the effects of exercise on muscle strength and mass, comparatively less attention has been given to the enhancement of muscle function through low‐intensity mind–body exercises [37, 38]. Compared with RT, BE produced greater improvement in body control, likely due to its emphasis on gentle stretching and joint mobilization, which enhance flexibility and range of motion. These characteristics, largely absent in traditional RT, may contribute to improved joint mobility, reduced stiffness and consequently better overall muscle function.

Moreover, the adaptability of BE to community and home settings enhances its accessibility and supports improved quality of life and independence among older adults. Health professionals and geriatric practitioners are therefore encouraged to incorporate BE into the care routines of community‐dwelling older adults and geriatric patients with sarcopenia as a safe, feasible and effective intervention.

### 4.1 Strengths and Limitations

This research represents the first RCT to utilize the SPPB scale as the primary observational index. We implemented strict quality control measures throughout the intervention. In particular, our study employed a qualified instructor to lead the sessions onsite, and this help ensured that participants adhered strictly to the exercise protocols. Although BE is well‐known for its gentle movements, simplicity and adaptability—distinct from other traditional Chinese *qigong* exercises such as Tai chi chuan—its application in the geriatric sarcopenia population remains limited. The findings of this study helped bridge this research gap by demonstrating the effectiveness of BE in enhancing limb function, thereby supporting its broader implementation in geriatric sarcopenia management and underscoring its potential clinical value in the prevention and treatment of sarcopenia.

Several limitations in this study should be acknowledged. First, the recruitment of only 90 subjects from the Affiliated Hospital of Chengdu University of TCM and its surrounding community, coupled with the predominance of female participants (85.56%) and the relatively small sample size, may have reduced the statistical power to detect subtle yet clinically meaningful differences between the intervention and control groups.

Second, outcome measurements were collected only at Weeks 12 and 24, which limited the ability to observe intermediate trends or short‐term fluctuations in response to the intervention. Additionally, some participants experienced difficulties maintaining consistent attendance at BE sessions due to external factors such as transportation issues or personal health conditions. Variability in adherence may have introduced bias, potentially leading to underestimation of the intervention's true effect on sarcopenia‐related outcomes. Future multicentre studies with larger and more diverse populations are warranted to enhance the generalizability of these findings.

Third, most participants were elderly hospital patients, with 63.33% presenting comorbid conditions such as osteoarthritis and muscular fibromyalgia, which may have affected their ability to perform the exercises as prescribed. This selective demographic may therefore not fully represent the broader population of older adults with sarcopenia. Future research should explore the efficacy of BE across varying severities of sarcopenia and investigate potential synergistic effects when combined with nutritional and other lifestyle interventions. Moreover, additional studies should examine the impact of BE on cognitive function and mental health among elderly individuals, given its mind–body integrative nature [39].

## 5 Conclusions

The present study demonstrated that regular practice of BE significantly enhances muscle strength, physical function and SMI of the lower limbs in older adults. These findings underscore the value of BE as an effective therapeutic modality for improving musculoskeletal health. Moreover, the results highlight BE as a safe, low‐intensity and accessible form of exercise suitable for the management of sarcopenia, with strong potential for integration into geriatric care settings. Given its simplicity, safety and adaptability, BE exercise is recommended as a complementary intervention in treatment and prevention programs that target sarcopenia among older adults.

## Funding

This work was supported by the National Natural Science Foundation of China (No. 82174345), the China Scholarship Council Foundation (No. 202208735003) and the Sichuan Cadre Health Research Project (CGY‐2022‐504 and CGY‐2023‐504).

## Conflicts of Interest

The authors declare no conflicts of interest.

## Supporting information


**Data S1:** Supporting Information.

**Data S2:** Supporting Information.

**Data S3:** Supporting Information.

## Data Availability

The data supporting the findings of this study are available from the corresponding author upon reasonable request. Deidentified data may be shared with researchers who meet the criteria for access to confidential data, provided that they submit a methodologically sound proposal and sign a data use agreement. The data will be available for sharing from the date of publication.
