# Supplementary material for: Efficacy of Baduanjin Exercise for Sarcopenia in Older Adults: A 24‐Week Randomized Controlled Trial
Source: J Cachexia Sarcopenia Muscle. 2025 Dec 10;16(6):e70163. doi: 10.1002/jcsm.70163 (PMC12695133; doi:10.1002/jcsm.70163)
Supplement: Supplementary file 1 — Data S1: Supporting Information. [file JCSM-16-e70163-s002.docx]

**Trial Protocol and Statistical Analysis Plan**

**Contents**

**1. Introduction — P2**

**2. Purposes — P3**

**3. Methods — P3**

**3.1 Participants**

**3.1.1 Diagnostic criteria**

**3.1.2 Inclusion criteria**

**3.1.3 Exclusion criteria**

**3.1.4 Suspension criteria**

**3.1.5 Elimination criteria**

**4. Sample size — P5**

**5. Participant — P5**

**6. Randomization and Blinding — P6**

**7. Interventions — P6**

**7.1 The Baduanjin Group**

**7.2 The Resistance training Group**

**7.3 Assessment of the day-to-day activities of the intervention**

**7.4 Exercise intensity assessment**

**7.5 Assessment of sports attendance**

**8. Measure — P8**

**8.1 Demographics**

**8.2 Primary outcome**

**8.3 Secondary outcomes**

**9. Quality control — P10**

**9.1 Quality control for researchers**

**9.2 Quality control of study participant enrolment**

**9.3 Quality control of the intervention process**

**9.4 Quality control of data management**

**10. Statistical analysis — P11**

**10.1 Power calculation**

**10.2 Data analyses**

**11. References — P12**

**Title:**

**The Efficacy of Baduanjin Exercise for Sarcopenia in the Elderly: A 24-Week Randomized Controlled Trial**

Institution of Research: Hospital of Chengdu University of Traditional Chinese Medicine

Principal Investigator: Bin Li

Institution of Ethical Review: Hospital of Chengdu University of Traditional Chinese Medicine

Date of Version: 26th July 2021

Contact Information: libin@cdutcm.edu.cn

1. **Introduction**

As demographic shifts alter the population composition, with a marked escalation in the elderly demographic, China is swiftly transitioning into an aged society. This shift has precipitated a substantial surge in the prevalence of age-associated maladies, presenting a formidable challenge to public health. During the senescence process, an inevitable decline occurs in the physiological integrity of organ systems. Skeletal muscle, an integral component of locomotion, serves as a vital repository for proteins and the principal entity for glycemic regulation. The deterioration in skeletal muscle quality and functionality is emblematic of the aging process. A prevalent condition among the elderly, characterized by faltering gait and diminished swiftness, was once deemed a natural aspect of aging. Contemporary research, however, suggests that these symptoms may be attributable to sarcopenia—a condition defined by the erosion of skeletal muscle mass, coupled with waning endurance, strength, and metabolic efficacy ^[1]^. The term ‘sarcopenia’ was coined by Rosenberg in 1989, denoting the age-related decrement in muscular mass and potency ^[2]^. The European Working Group on Sarcopenia in Older People (EWGSOP) refined this definition in 2010, describing it as a progressive and pervasive reduction in skeletal muscle mass and strength, which precipitates a decline in physical activity and functionality ^[3]^. In a landmark decision in October 2016, sarcopenia was officially recognized and codified within the International Classification of Diseases (ICD-10) ^[4]^.

Currently, the etiology of sarcopenia remains a subject of global debate, with consensus yet to be reached. The scholarly community widely acknowledges that sarcopenia in the elderly stems from a confluence of factors, rather than a singular cause. As a chronic condition that accelerates the aging process, the pathogenesis of sarcopenia is intricate and multifaceted. Aging and sedentary lifestyles rank among the primary contributors to sarcopenia, leading to a reduction in skeletal muscle mass, fibrotic infiltration, and adipose tissue replacement within muscle fibers, as well as atrophy of type II fibers and compromised neural control over muscle function ^[5]^. Research from both domestic and international sources indicates that elderly individuals with insufficient physical activity constitute a high-risk demographic for the development of sarcopenia ^[6]^. As the body ages, there is a notable decline in skeletal muscle mass and muscular strength among the elderly, which restricts their physical activity. This limitation not only impedes normal physical exertion but, over time, also precipitates a decline in health and functional capacity.

In 2010, the European Working Group on Sarcopenia in Older People (EWGSOP) delineated sarcopenia into three distinct stages: pre-sarcopenia, sarcopenia, and severe sarcopenia. Pre-sarcopenia is characterized solely by a reduction in muscle mass. Sarcopenia extends beyond this to include a decline in muscle strength or function. This stratification is instrumental in formulating clinical intervention strategies and objectives.

The pathogenesis of sarcopenia in the elderly is attributed to both intrinsic and extrinsic factors. Intrinsic factors encompass age-related skeletal muscle atrophy, structural alterations within the musculature, mitochondrial dysfunction, degeneration of motor nerves, and hormonal decline. Extrinsic factors include malnutrition and a lack of physical activity [7][8][9]. Given the deleterious side effects associated with hormone therapy, its application in clinical settings is constrained. Consequently, nutritional and exercise interventions have emerged as the cornerstone treatments for sarcopenia, representing fundamental strategies in managing this condition in the elderly population ^[9]^.

Traditional Chinese medicine (TCM) health care practices, such as Baduanjin and Tai chi Chuan, are quintessential to China’s rehabilitative exercise regimen. These ancient techniques are revered for their capacity to harmonize the internal organs, meridians, and the flow of qi and blood, thereby fostering spiritual well-being and mental fortitude. Each gong method is distinct; for instance, Tai chi Chuan is known for its intricate and elaborate movements, which may pose a challenge for the layperson to master.

In contrast, Baduanjin stands out as a holistic and self-contained fitness system that has been cherished for generations. It is celebrated for its body-strengthening effects and is a staple in the realm of traditional health-promoting exercises. Baduanjin aligns seamlessly with the principles of kinesiology and physiology ^[10]^, offering an accessible, safe, and efficacious workout that transcends common barriers such as time, location, and cost. It is readily embraced by patients due to its ease of learning and adaptability to various conditions. Moreover, Baduanjin’s philosophy of balancing yin and yang to heal the spirit further enhances its therapeutic value, making it particularly beneficial for patient rehabilitation. As a form of low- to medium-intensity aerobic exercise ^[11]^, Baduanjin is ideally suited to the national context of China. Research by Zou L et al. ^[12]^ substantiates the efficacy of Baduanjin exercises, highlighting improvements in quality of life, sleep patterns, trunk stability, grip strength, and trunk flexibility. This evidence underscores the significance of Baduanjin as a culturally congruent and scientifically validated approach to health and rehabilitation in China.

1. **Purposes**

Utilizing the Short Physical Performance Battery (SPPB) as a metric, this investigation aims to scrutinize the clinical outcomes of a consistent regimen of Baduanjin exercises in geriatric patients diagnosed with sarcopenia. Concurrently, it endeavors to assess the impact of the Baduanjin technique, rooted in traditional Chinese medicine qigong, on the life quality and safety of these patients. The objective is to devise a standardized Baduanjin exercise protocol tailored for sarcopenia, thereby pioneering an exercise rehabilitation paradigm that resonates with China’s national ethos. This study aspires to furnish a robust theoretical framework that will underpin the practical application and widespread dissemination of this approach in the management of sarcopenia among the elderly. Furthermore, it seeks to contribute a scholarly foundation for the endorsement and implementation of Baduanjin in the realm of geriatric sarcopenia.

1. **Methods**

**3.1 Participants**

**3.1.1 Diagnostic criteria**

The diagnostic criteria set forth by the Asian Working Group for Sarcopenia ^[13]^ are as follows:

- A: SMI (Skeletal Muscle Index): This is determined using Bioelectrical Impedance Analysis (BIA) and should be less than or equal to 7.0 kg/m^2^ for males and less than or equal to 5.7 kg/m^2^ for females.
- B: Muscle strength (Handgrip Strength): The strength threshold is less than 28 kg for males and less than 18 kg for females.
- C: Physical performance

6-metre walk: <1.0 m/s

or 5-me chair stand test: ≥12 s

or Short Physical Performance Battery: ≤9

A diagnosis of sarcopenia is confirmed when both criteria A and B or A and C are met. It's important to note that these criteria are specifically tailored to the Asian population and take into account the distinct body composition profiles prevalent in this demographic.

**3.1.2 Inclusion criteria**

- Participants should be elderly individuals aged between 60 and 84 years, who conform to the established diagnostic benchmarks for sarcopenia.
- Candidates must be amenable to engaging in comprehensive body composition analysis, handgrip strength assessment, 6-meter walk time evaluation, and completing pertinent questionnaires.
- Prospective participants ought to possess the ability to ambulate independently, whether unassisted or utilizing supportive devices.
- Individuals should demonstrate the capacity to safely execute exercise regimens as prescribed by medical professionals.
- Participants must express their willingness to partake in the study, adhere to the randomized intervention protocol, and provide signed informed consent.

**3.1.3 Exclusion criteria**

- Individuals exhibiting a 6-meter walking speed of less than 1.0 m/s, or those who require 12 seconds or longer to complete 5 sit-ups, or have an SPPB (Short Physical Performance Battery) score greater than 9 points.
- Patients afflicted with severe pathologies that influence muscle and bone metabolism, including but not limited to renal disease, malignant tumors, cerebrovascular accidents, advanced liver disease, severe diabetes mellitus, thyroid disorders, and parathyroid diseases.
- Those currently undergoing treatment with medications known to impact muscle and bone metabolism, such as glucocorticoids, androgens, oestrogens, thyroid hormones, among others.
- Individuals who have a consistent routine of physical activity, defined as engaging in high-intensity physical activity for at least 15 minutes daily, or participating in plyometric training at least twice weekly in the 12-week preceding enrollment.
- Participants with significant cognitive deficits, as evidenced by a score below 20 on the Simple Intelligence Scale.

**3.1.4 Suspension criteria**

- Should a participant express a desire to discontinue their involvement in the study at any point, their request will be honored, and they will be withdrawn from the clinical trial.
- Participants who are untraceable or fail to attend scheduled follow-up assessments will be considered lost to follow-up and subsequently withdrawn from the study.
- In the event that a participant develops new comorbidities, complications, or undergoes physiological changes during the study that render them unsuitable for continued intervention, they will be removed from the trial.
- If a participant’s health status significantly worsens during the study, potentially leading to a critical event, and the supervising physician deems it necessary to halt the clinical trial for the individual’s safety, the participant will be withdrawn from the study.

**3.1.5 Elimination criteria**

- Participants who are subsequently found to not fulfill the inclusion criteria after the commencement of the study will be excluded.
- Subjects who decline to participate in the prescribed examination and assessment protocol during the study will be withdrawn.
- Participants demonstrating suboptimal adherence, with cumulative exercise time falling below 80% of the prescribed amount, or those who consume medication that influences muscle or bone metabolism during the study period, will be considered non-compliant and thus removed from the trial.

1. **Sample size**

The prevalence of functional falls and fractures is a significant clinical concern and a severe consequence for patients with sarcopenia, stemming from the progressive loss of muscle mass and function. The Simple Physical Performance Measurement Scale (SPPB) serves as a pivotal efficacy indicator in this context. Drawing from extant literature ^[14]^, a 24-week exercise intervention in elderly patients with a heightened risk of falls yielded an SPPB score of 9.83 ± 1.85 in the experimental group, compared to 8.39 ± 2.52 in the control group. Employing a two-sided test with an alpha level (α) of 0.05 and a power (test efficacy) of 0.9, the calculated sample size for a single group is n = 35.6, which approximates to 36 participants. Consequently, the combined sample size for both groups amounts to 72 individuals. Factoring in a dropout rate of 20%, the total sample size required for this study is 90 cases, equating to 45 participants per group.

1. **Participant**

The proposed study aims to enroll 90 elderly patients with sarcopenia who visited the Hospital of Chengdu University of Traditional Chinese Medicine between July 2022 to August 2023. The process for inclusion involves a meticulous screening of candidates at the outpatient clinic. Potential participants are to be thoroughly informed about the study’s objectives, procedures, and potential risks. Following this educational dialogue, those who meet the study criteria and express a willingness to participate will be asked to provide informed consent and sign the corresponding consent form.

1. **Randomization and Blinding**

The random allocation sequence was delegated to a specialized statistical analyst, who utilized the SAS 9.4 software (SAS, USA) to generate a series of random numbers ranging from 1 to 90. Subsequently, the analyst enumerated the eligible participants in sequential order of their enrollment and allocated them randomly in a balanced 1:1 ratio to either the Baduanjin exercise intervention cohort—hereinafter referred to as the ‘Baduanjin group’—or the resistance training comparator arm—hereafter denoted as the ‘control group’. The integrity of the randomization sequence was preserved by the study administrator, who remained uninvolved in participant recruitment, outcome evaluation, and statistical analysis. Upon completion of all initial assessments, participants were apprised of their group assignment via telephone.

Due to the nature of the intervention, it was impracticable to blind the study participants. Consequently, blinding was confined to the outcome assessment, data management, and statistical analysis phases. In lieu of explicit group identifiers, the designations ‘A’ and ‘B’ were employed on the case report forms to maintain anonymity. An independent statistical expert, external to the participant cohort, employed SAS software to create the blind base—a table of random numbers—which was subsequently safeguarded by the study administrator. The statistician tasked with data analysis remained uninformed about the specific intervention programs administered to each group. This blinding was only disclosed subsequent to the completion of all statistical analyses.

1. **Interventions**

**7.1 The Baduanjin Group**

In conjunction with health education initiatives, the Baduanjin training is conducted at a designated time and location. Each session is facilitated by an instructor with a minimum of five years’ experience in teaching Baduanjin. The training adheres to the Baduanjin standards promulgated by the State General Administration of Sports in 2006 ^[15]^. The sequence comprises a preparatory stance followed by a series of ten movements:

- Preparatory Posture
- Holding Up The Sky With Both Hands To Regulate The Sanjiao
- Draw The Bow On Both Sides As If To Shoot A Vulture
- Stretch Arms Asymmetrically To Enhance Spleen And Stomach Function
- Look Back To Treat Five Strains And Seven Impairments
- Sway The Head And Buttocks To Subdue The Heat Fire
- Touch Feet To Strngthen Kidneys And Waist
- Rise Up On Toes And Land On Heels Repeatedly To Cure Disease
- Clench The Fist And Open Eyes Wide To Enhance Vitality
- Closing Posture

The regimen is scheduled three times weekly, with each session lasting 60 minutes. This includes 15 minutes for warm-up activities, 30 minutes dedicated to the Ba Duan Jin exercises, and 15 minutes for cool-down activities, spanning a total duration of 24 weeks.

**7.2 The Resistance training Group**

The educational component of the study on sarcopenia in the elderly is structured to provide sessions once every four weeks, each lasting no less than 30 minutes. These sessions are designed to impart knowledge on the risk factors, adverse consequences, and preventive measures associated with sarcopenia. An example of such preventive measures includes adherence to the dietary guidelines for the elderly, as revised in 2016 by the Chinese Nutrition Society, which emphasizes rational nutrition, balanced diets, and the cultivation of healthy living and eating habits. In terms of physical intervention, the study incorporates a resistance training program tailored to the geographic location of each sub-centre. The regimen consists of 60-minute sessions conducted three times a week over a 24-week period. Each training session is segmented into a 15-minute warm-up, followed by 30 minutes of resistance exercises—including Wall squat, Wall push-up, Forward lunge, Reverse triceps push-up, and Wall-supported calf raise—and concludes with 15 minutes of cool-down activities.

**7.3 Assessment of the day-to-day activities of the intervention**

To mitigate the potential confounding impact of routine physical activities on the efficacy of the exercise intervention, participants are required to meticulously document their daily activities at the conclusion of weeks 1, 12 and 24 post-intervention commencement. This documentation encompasses the average daily activity and its corresponding duration over the preceding week. The catalog of activities spans a spectrum from sleep and sedentary work to activities of low, medium, and high intensity. The collected data, encapsulated in activity fact sheets, are subsequently retrieved via telephone follow-ups conducted by the research team.

**7.4 Exercise intensity assessment**

The assessment of exercise intensity in your study is based on the percentage of maximum heart rate. Continuous heart rate monitoring is conducted throughout the exercise session using a Mio heart rate monitor (Mio Alpha II, USA). At the conclusion of the exercise, the average heart rate output is determined. This is done by measuring the heart rate three times for the same individual and calculating the mean value. The percentage of maximal heart rate is calculated using the following formula:

- % of maximal heart rate=(measured heart rate / 220−age)×100%

The target exercise intensity is set to moderate or upper moderate, which corresponds to 60%-80% of the maximum heart rate value.

**7.5 Assessment of sports attendance**

The protocol for tracking attendance in your study is well-defined. Intervention supervisors are responsible for recording attendance at each training session. Participants who engage in the training for less than 20 minutes are marked as absent.

The attendance rate is calculated at the conclusion of the intervention period using the formula:

- % attendance=(number of training sessions attended / total number of training sessions planned)×100%

1. **Measure**

**8.1 Demographics**

For the comprehensive assessment of participants in your study on sarcopenia, the following data should be meticulously collected:

- Demographic Data: This includes the participant’s name, gender, date of birth, education level, weight, height (or BMI), ethnicity, occupation, and contact address.
- General Physical Examination: Vital signs such as heart rate, pulse, respiration, and blood pressure should be systematically recorded.
- Medical and Social History:
- Current Medical History: Document any present health conditions.
- Co-morbidities and Medications: List any concurrent illnesses and current medications.
- Allergies: Note any known allergies.
- Family History: Include any relevant hereditary health issues.
- Smoking and Alcohol History: Record past and present tobacco and alcohol use.
- Additional Relevant Information: Gather details on any falls within the past year, hand dexterity (sharp hands), sleeping habits, hobbies, personality traits, social activities, and other lifestyle factors that may influence the condition.

**8.2 Primary outcome**

The Short Physical Performance Battery (SPPB) is indeed a widely recognized assessment tool for evaluating the physical function of older adults. It encompasses three key components:

- Repeated Chair Stands: This task measures lower body strength by timing how long it takes to stand from a seated position and sit down again, repeated five times.
- Balance Tasks: These tasks assess static balance through three progressively challenging positions—side-by-side stand, semi-tandem stand, and tandem stand—each held for 10 seconds.
- Gait speed: This component evaluates walking speed over a short distance as a measure of gait speed and overall mobility.

Each task is scored individually, and the scores are then aggregated to yield an overall performance score ranging from 0 (indicating the poorest function) to 12 (representing the best function). Higher SPPB scores correlate with better physical function and mobility in older adults.

**Table 1. Simple Physical Performance Battery (SPPB)**

| Test items | | Criteria for judging | score |
| --- | --- | --- | --- |
| Balance test | stand on one foot | Hold on 10s. | 1 |
|  | half-fore-and-aft stance | Failure to hold 10s | 0 |
|  | front-and-back stance | Hold on 10s. | 1 |
| 4 meters walking time | | Failure to hold 10s | 0 |
|  |  | Hold on 10s. | 2 |
|  |  | Hold on 3s to 9.99s. | 1 |
|  |  | Hold on <3s | 0 |
|  |  | Time <4.82s | 4 |
|  |  | Time 4.82s to 6.20s | 3 |
|  |  | Time 6.21s to 8.70s | 2 |
|  |  | Time >8.70s | 1 |
|  |  | unfinished | 0 |
| Repeat 5 sit-up test | Before the test (without hands) | Time <11.20s | 4 |
|  | Repeat 5 times | Time 11.20s to 13.69s | 3 |
|  |  | Time 13.70s to 16.69s | 2 |
|  |  | Time >16.70s | 1 |
|  |  | Takes more than 60s or cannot be completed | 0 |
| Total score | | |  |

**8.3 Secondary outcomes**

The methodology for assessing the efficacy of the intervention on elderly patients with sarcopenia is comprehensive and multi-faceted, involving the following measures:

- Body Composition Analysis: Utilizing the DBA-510 Body Composition Analyzer, participants' body weight, body water, fat mass, fat percentage, lean body weight, BMI, and segmental muscle mass of the trunk, upper, and lower limbs will be evaluated through bioelectrical impedance analysis.
- Handgrip Strength Measurement: The JAMAR electronic grip strength meter will be employed to gauge upper limb muscle strength. Participants will perform the test standing, with arms at their sides, elbows bent at 90°, and forearms supinated, exerting maximum grip strength. The highest value from four measurements (two per side) will be recorded.
- 6-Meter Walk Test: This test assesses muscle performance by having participants walk 6 meters at their usual pace. The walking speed is calculated based on the time taken to complete the distance. The test is conducted twice, and the average time and speed are noted.
- Cognitive Function Assessment: The 30-item Montreal Cognitive Assessment (MoCA, Changsha version) will be used to measure global cognitive function. This instrument is tailored for Chinese populations and scores cognitive function across multiple domains on a scale from 0 to 30, with higher scores indicating better cognitive function.
- Incidence of Falls Tracking: Participants will maintain a "fall calendar" diary to document any falls and related medical consultations monthly. This includes accidental landings on the floor or ground or collisions with objects. The collection of fall data commences with the first intervention class and continues until the intervention's conclusion or until a participant's withdrawal, death, or loss to follow-up.

1. **Quality control**

**9.1 Quality control for researchers**

The recruitment process for the study was meticulously orchestrated to ensure the selection of appropriate subjects. Specialized recruiters conducted screenings with stringent adherence to the predetermined inclusion and exclusion criteria. Subjects who satisfied all inclusion criteria and none of the exclusion criteria were systematically enrolled and assigned a unique study number reflecting their sequential order of inclusion. Conversely, individuals who failed to meet the necessary criteria were excluded from the study. The recruiters maintained detailed records of each exclusion, documenting both the quantity and the specific reasons for disqualification.

**9.2 Quality control of study participant enrolment**

The recruitment protocol for the study was executed with precision by specialized recruiters who adhered strictly to the established inclusion and exclusion criteria. Candidates who fulfilled all the inclusion criteria while not meeting any of the exclusion criteria were systematically included in the study and sequentially assigned a study number. Conversely, individuals who did not meet the qualifying criteria were excluded. The recruiters meticulously documented each exclusion, noting the total number of exclusions along with the specific reasons for each disqualification.

**9.3 Quality control of the intervention process**

Throughout the duration of the intervention, certified coaches were mandated to demonstrate uniform and standardized movements, providing continuous instruction to participants and promptly rectifying any deviations in form. Concurrently, researchers meticulously oversaw each training session, documenting attendance on-site. They calculated the attendance rate post-intervention and instituted a protocol whereby participants unable to attend centralized sessions were required to request and log their absence in advance. This measure was implemented to guarantee adherence to the prescribed exercise regimen of one hour, thrice weekly. To mitigate the potential disruptions caused by inclement weather conditions such as rain or snow, indoor facilities like community centers were strategically selected as the venues for the intervention.

**9.4 Quality control of data management**

To uphold the integrity of the data collection process, researchers implemented standardized methodologies aimed at minimizing bias. This involved meticulous storage of original documents, which were then individually verified post-assessment to confirm data completeness and accuracy. Such diligence ensures that the collected information is both reliable and valid for subsequent analysis.

1. **Statistical analysis**

**10.1 Power calculation**

The prevalence of falls and fractures due to the diminution of muscular mass and function underscores the clinical urgency and grave repercussions associated with sarcopenia. Drawing upon empirical evidence from a preceding trial targeting older adults predisposed to falls ^[14]^, the Short Physical Performance Battery (SPPB) is employed as the primary efficacy index. Utilizing a two-sided test with a power of 90% and a significance level of 5%, the requisite sample size for a single intervention group is deduced to be approximately 36. Accounting for an anticipated dropout rate of 20%, each group necessitates 45 participants diagnosed with sarcopenia.

**10.2 Data analyses**

Following an intention-to-treat (ITT) protocol, those whose cumulative time of exercise reaches 80% of the amount to be completed and not higher than 120% of that will be classified into per protocol set (PPS). Categorical variables will be presented as percentages or frequencies. As for continuous variables, normally distributed data will be described by mean ± standard deviation, while non-normally distributed data will be described using median [interquartile range (IQR)]. Baseline demographic descriptors and outcome measures wil be compared across groups by using analysis of variance for continuous variables and the $\chi$*^2^* (or Fisher exact) test for categorical variables. Within the scope of this investigation, the Generalized Estimating Equations (GEE) framework was employed to evaluate the impact of Baduanjin exercise on sarcopenia among the elderly cohort. GEE is a robust statistical approach designed to analyze correlated data or repeated measurements over time, rendering it particularly adept at accommodating incomplete datasets and multiple observational instances.Initially, an exhaustive data cleansing and encoding procedure will be undertaken to ascertain the precision and integrity of the dataset. Continuous variables will undergo a distributional analysis to discern the necessity of transformation to align with the normal distribution assumption. Categorical variables will be delineated through frequencies and percentages.The GEE model will incorporate temporal factors as a repeated measures variable, alongside other pertinent covariates that may influence the outcomes, such as age, gender, and baseline muscle mass index. The inclusion of interaction terms pertinent to time will facilitate an in-depth assessment of the longitudinal effects of Baduanjin exercise on sarcopenia. The model’s fit will be gauged using the Quasi-likelihood under the Independence model Criterion (QIC), with preference given to the model demonstrating the minimal QIC value. Additionally, a thorough examination of model residuals will be conducted to validate the adherence to underlying assumptions.Two-sided P values of less than 0.05 will be considered statistically significant. Analyses will be conducted using SPSS version 23.0 (IBM Corp) or GraphPad Prism version 7.0 (GraphPad Software).

**Table 2. Schedule of trial procedures**

| **Period** | **Screening** | **Intervention period** | | |
| --- | --- | --- | --- | --- |
| **Time point** | **0 wk** | **0 wk** | **12 wk** | **24 wk** |
| **Enrollment** | **×** |  |  |  |
| Eligibility screening | **×** |  |  |  |
| Informed consent |  | **×** |  |  |
| **Allocation** |  |  |  |  |
| **Intervention** |  |  |  |  |
| Non-sarcopenia control |  | **×** |  |  |
| Baduanjin exercise |  | **×** | **×** | **×** |
| Resistance training |  | **×** | **×** | **×** |
| **Assessments** |  |  |  |  |
| Physical function |  | **×** | **×** | **×** |
| Body composition |  | **×** | **×** | **×** |
| Handgrip strength |  | **×** | **×** | **×** |
| 6 m walking test |  | **×** | **×** | **×** |
| Cognitive function |  | **×** | **×** | **×** |
| Incidence of falls |  | **×** |  | **×** |

1. **References**
2. Morley JE. Sarcopenia:diagnosis and treatment[J]. *J Nutr Health Aging,* 2008, 12: 452-456.
3. Fielding RA, Vellas B, Evans WG. Sarcopenia: an undiagnosed condition in older adults. Current consensus definition: prevalence, etiology, and consequences. International working group on sarcopenia[J]. *J Am Med Dir Assoc*. 2011, 12: 249-256.
4. Cruz-Jentoft AJ, Baeyens JP, Bauer LM. Sarcopenia: European consensuson definition and diagnosis: Report of the European Working Group on Sarcopenia in Oder People[J]. *Age Ageing*, 2010, 39(4): 412-423.
5. Cao Li MD, JE. Morley MB. Sacropenia is recognized as an independent Condition by an International Classification of Disease, Tenth Revision, Clinical Modification(ICD-10) Code[J]. 2016, (17): 675-677.
6. Murton AJ. Muscle protein turnover in the elderly and its potential contribution to the development of sarcopenia[J]. *Proc Nutr Soc*, 2015:74(4): 387-396.
7. Yuwei Lin, Guiyu Qian, Meizhi Xu. Causes and effects of sarcopenia in the elderly[J].*Tertiary sports*, 2010.10, (110): 85-91.
8. Fielding RA, Vellas B, Evans WJ, et al.Sarcopenia: an undiagnosed condition in older adults.Current consensus definition: prevalence, etiology, and consequences[J]. *J Am Med Dir Assoc*, 2011, 12: 249-256.
9. Chen LK, Liu LK, Woo J, et al. Sarcopenia in Asia: consensus report of the Asian Working Group for Sarcopenia[J]. *J Am Med Dir Assoc*,2014,15:95-101.
10. Forbes SC, Little JP, Candow DG. Exercise and nutritional interventions for improving aging muscle health[J]. *Endocrine*, 2012, 42: 29-38.
11. Biying Yang. Application of the eight-duanjin method of tonifying the lungs, spleen and kidneys in the adjuvant therapy of patients with COPD[J]. *International Journal of Nursing*, 2016, 35(17): 2357-2359.
12. Li Jin, Qinbo Xue, Ran Li, et al. A comparative study of the energy expenditure characteristics of Ba Duan Jin and the ninth set of broadcasting gymnastics[J].*Chinese Journal of Sports Medicine*, 2015, 34 (6): 588-591.
13. Zou L, SasaKi JE, Wang H, et al. A Systematic Review and eta-Analysis Baduanjin Qigong for Health Benefits:randomized controlled trials[J]. *Evid Based Complement Alternat Med*, 2017: 1-17.
14. Chen LK, Woo J, Assantachai P, et al. Asian Working Group for Sarcopenia: 2019 Consensus Update on Sarcopenia Diagnosis and Treatment[J]. *J Am Med Dir Assoc*. 2020;21(3):300-307.e2.
15. Li F, Harmer P, Fitzgerald K, Eckstrom E, Akers L, Chou LS, et al. Effectiveness of a therapeutic tai Ji Quan intervention vs a multimodal exercise intervention to prevent falls among older adults at high risk of falling: a randomized clinical trial[J]. *JAMA Intern Med*. 2018;178 (10):1301–10.
16. China HQmcogaoso. Health Qigong: Baduanjin[M]. Beijing: *People’s Sports Publishing House of China*; 2009.
