# Supplementary material for: Efficacy of Baduanjin Exercise for Sarcopenia in Older Adults: A 24‐Week Randomized Controlled Trial
Source: J Cachexia Sarcopenia Muscle. 2025 Dec 10;16(6):e70163. doi: 10.1002/jcsm.70163 (PMC12695133; doi:10.1002/jcsm.70163)
Supplement: Supplementary file 2 — Data S2: Supporting Information. [file JCSM-16-e70163-s001.docx]

**Table S1.Inclusion criteria, Exclusion criteria and Shedding Criteria of program**

| **Inclusion criteria** | | **Exclusion criteria** | **Shedding Criteria** |
| --- | --- | --- | --- |
| Baduanjin Group | Resistance training group | - 6m walking speed ≥1.2 m/s, or 5 times sit-up time ≤12s, or SPPB＞9 score. - Patients suffering from diseases that seriously affect muscle and bone metabolism, such as renal disease, malignant tumor, cerebrovascular accident, serious liver disease, serious renal disease, serious diabetes mellitus, thyroid disease, parathyroid disease, and so on. - Patients who are taking drugs that affect muscle and bone metabolism, such as glucocorticoids, androgens, estrogens, thyroid hormones, and so on. - Plainly have the habit of sports and exercise: 15 minutes or more of high-intensity physical activity per day, or two or more times per week of plyometric training in the 3 months before enrollment. - Combined severe cognitive impairment (score below 20 on the Simple Intelligence Scale). | - During the study period, the subject is unwilling to continue with follow-up and requests to stop the clinical trial. - During the study period, the subject develops certain comorbidities, complications, or special physiological changes that may make him/her unsuitable to continue the intervention or participate in the trial. - During the study period, the subject's condition continues to deteriorate and there is a possibility of a critical event, and the physician in charge of the study determines that the clinical trial should be stopped. |
| - Elderly people aged 60-84 years old who meet the diagnostic criteria for sarcopenia. - Able to complete body composition analysis, grip strength measurement, 6m walking time measurement and questionnaire. - Be able to walk independently, with or without assistive devices. - Be able to safely complete the exercises recommended by healthcare professionals.   Agree to participate in the study, accept and implement the intervention program after randomization, and sign the informed consent form. | |  |  |

**Table S2. Simple Physical Performance Battery (SPPB)**

| Test items | | Criteria for judging | score |
| --- | --- | --- | --- |
| Balance test | stand on one foot | Hold on 10s. | 1 |
|  | half-fore-and-aft stance | Failure to hold 10s | 0 |
|  | front-and-back stance | Hold on 10s. | 1 |
| 4 meters walking time | | Failure to hold 10s | 0 |
|  |  | Hold on 10s. | 2 |
|  |  | Hold on 3s to 9.99s. | 1 |
|  |  | Hold on <3s | 0 |
|  |  | Time <4.82s | 4 |
|  |  | Time 4.82s to 6.20s | 3 |
|  |  | Time 6.21s to 8.70s | 2 |
|  |  | Time >8.70s | 1 |
|  |  | unfinished | 0 |
| Repeat 5 sit-up test | Before the test (without hands) | Time <11.20s | 4 |
|  | Repeat 5 times | Time 11.20s to 13.69s | 3 |
|  |  | Time 13.70s to 16.69s | 2 |
|  |  | Time >16.70s | 1 |
|  |  | Takes more than 60s or cannot be completed | 0 |
| Total score | | |  |

*Throughout the process, if the score is 0, the test is stopped and moved to the next item.

**Table S3 Baseline Outcomes of the Participants**

|  | ALL(n=90) | BG(n=45) | RG(n=45) |
| --- | --- | --- | --- |
|  | Mean ± SD | | |
| **Short Physical Performance Battery (SPPB)** | 7.44 ± 1.50 | 7.24 ± 1.38 | 7.64 ± 1.60 |
| *Balance* | 2.39 ± 0.82 | 2.38 ± 0.65 | 2.40 ± 0.96 |
| *Lower limb strength* | 2.49 ± 0.94 | 2.29 ± 0.97 | 2.69 ± 0.87 |
| *Gait speed* | 2.57 ± 0.81 | 2.58 ± 0.72 | 2.56 ± 0.89 |
| **Skeletal muscle index (SMI) (kg/m^2^)** | 5.60 ± 0.40 | 5.61 ± 0.41 | 5.59 ± 0.39 |
| *Arm muscle mass (left) (kg)* | 1.99 ± 0.43 | 1.95 ± 0.45 | 2.03 ± 0.41 |
| *Arm muscle mass (right) (kg)* | 1.97 ± 0.35 | 1.80 ± 0.29 | 2.15 ± 0.32 |
| *Leg muscle mass (left) (kg)* | 5.01 ± 0.58 | 5.12 ± 0.67 | 4.89 ± 0.45 |
| *Leg muscle mass (right) (kg)* | 5.17 ± 0.63 | 5.21 ± 0.69 | 5.13 ± 0.58 |
| **Handgrip strength** |  |  |  |
| *Hand(left) (kg)* | 15.76 ± 1.51 | 15.44 ± 2.02 | 16.07 ± 0.59 |
| *Hand(right) (kg)* | 16.37 ± 0.98 | 16.2 ± 1.1 | 16.53 ± 0.82 |
| **6-meter walk test (m/s)** | 0.98 ± 0.14 | 0.98 ± 0.14 | 0.99 ± 0.15 |

**Table S4 12^th^ Week Outcomes of the Participants**

|  | ALL(n=90) | BG(n=45) | RG(n=45) |
| --- | --- | --- | --- |
|  | Mean ± SD | | |
| **Short Physical Performance Battery (SPPB)** | 8.8 ± 1.32 | 9.1 ± 1.17 | 8.5±1.4 |
| *Balance* | 2.96 ± 0.49 | 3.1 ± 0.44 | 2.83 ± 0.5 |
| *Lower limb strength* | 2.93 ± 0.65 | 2.93 ± 0.66 | 2.93 ± 0.66 |
| *Gait speed* | 2.91 ± 0.72 | 3.08 ± 0.66 | 2.75 ± 0.74 |
| **Skeletal muscle index (SMI) (kg/m^2^)** | 5.87 ± 0.47 | 6 ± 0.52 | 5.74 ± 0.38 |
| *Arm muscle mass (left) (kg)* | 2.02 ± 0.44 | 2.03 ± 0.44 | 2.02 ± 0.45 |
| *Arm muscle mass (right) (kg)* | 2.04 ± 0.37 | 1.87 ± 0.32 | 2.2 ± 0.34 |
| *Leg muscle mass (left) (kg)* | 5.25 ± 0.69 | 5.47 ± 0.85 | 5.04 ± 0.38 |
| *Leg muscle mass (right) (kg)* | 5.44 ± 0.7 | 5.58 ± 0.88 | 5.3 ± 0.43 |
| **Handgrip strength** |  |  |  |
| *Hand(left) (kg)* | 17.19 ± 1.7 | 17.12 ± 1.9 | 17.26 ± 1.51 |
| *Hand(right) (kg)* | 17.09 ± 1.18 | 17.49 ± 1.31 | 16.69 ± 0.87 |
| **6-meter walk test (m/s)** | 1.01 ± 0.14 | 1.01 ± 0.14 | 1.01 ± 0.14 |

**Table S5 24^th^ Week Outcomes of the Participants**

|  | ALL(n=90) | BG(n=45) | RG(n=45) |
| --- | --- | --- | --- |
|  | Mean ± SD | | |
| **Short Physical Performance Battery (SPPB)** | 9.82 ± 1.31 | 10.56 ± 0.97 | 9.05 ± 1.16 |
| *Balance* | 3.18 ± 0.76 | 3.54 ± 0.55 | 2.82 ± 0.77 |
| *Lower limb strength* | 3.3 ± 0.61 | 3.46 ± 0.55 | 3.13 ± 0.62 |
| *Gait speed* | 3.34 ± 0.64 | 3.56 ± 0.6 | 3.11 ± 0.61 |
| **Skeletal muscle index (SMI) (kg/m^2^)** | 5.96 ± 0.48 | 6.15 ± 0.53 | 5.77 ± 0.32 |
| *Arm muscle mass (left) (kg)* | 2.09 ± 0.43 | 2.05 ± 0.45 | 2.12 ± 0.41 |
| *Arm muscle mass (right) (kg)* | 2.07 ± 0.37 | 1.9 ± 0.34 | 2.24 ± 0.33 |
| *Leg muscle mass (left) (kg)* | 5.36 ± 0.71 | 5.64 ± 0.83 | 5.06 ± 0.39 |
| *Leg muscle mass (right) (kg)* | 5.52 ± 0.73 | 5.74 ± 0.88 | 5.3 ± 0.45 |
| **Handgrip strength** |  |  |  |
| *Hand(left) (kg)* | 17.27 ± 1.67 | 17.38 ± 1.97 | 17.17 ± 1.31 |
| *Hand(right) (kg)* | 17.37 ± 1.35 | 17.94 ± 1.48 | 16.79 ± 0.88 |
| **6-meter walk test (m/s)** | 1.03 ± 0.18 | 1.03 ± 0.15 | 1.04 ± 0.2 |

**
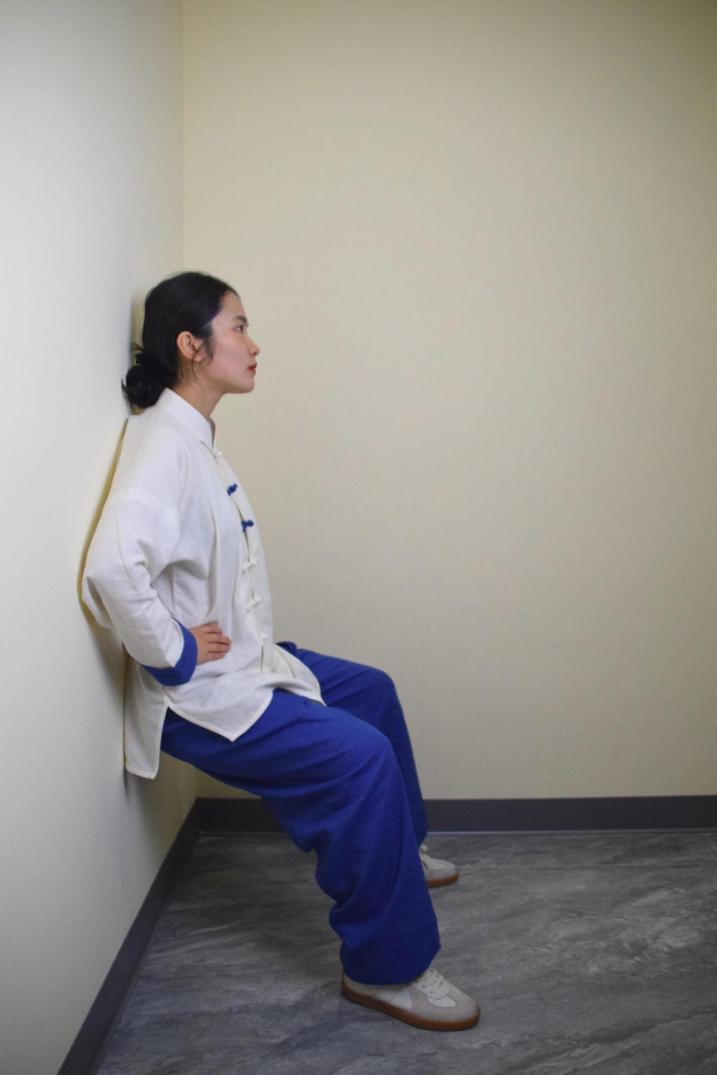
**

**Figure S1. Wall squat**

**
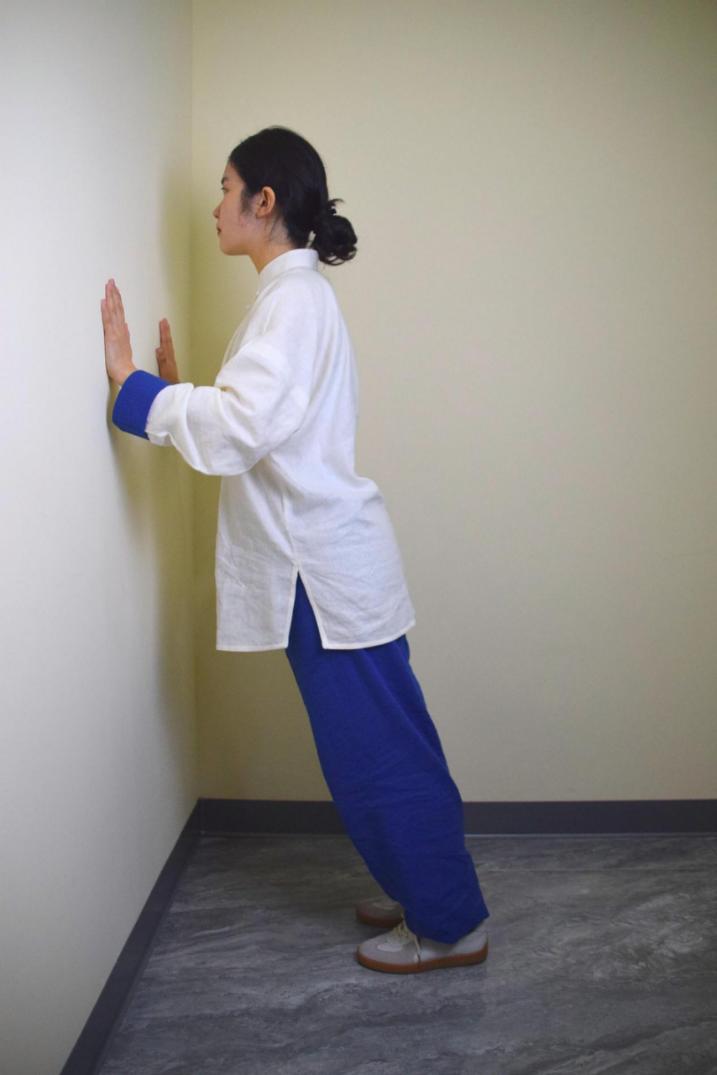
**

**Figure S2. Wall push-up**

**
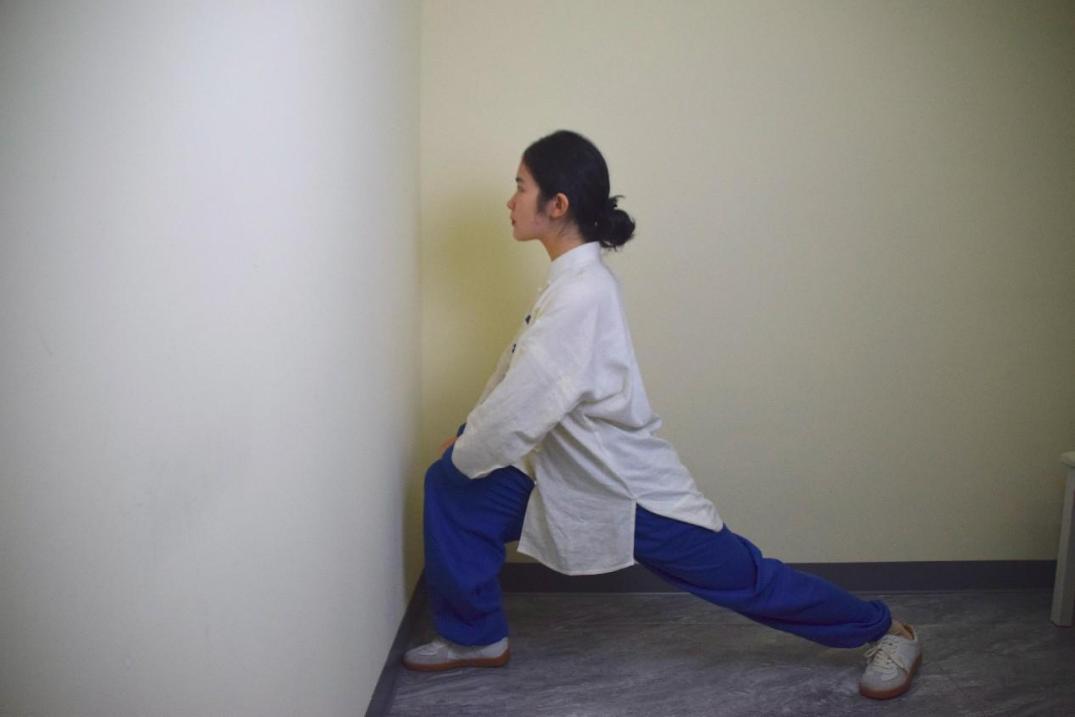
**

**Figure S3. Forward lunge**

**
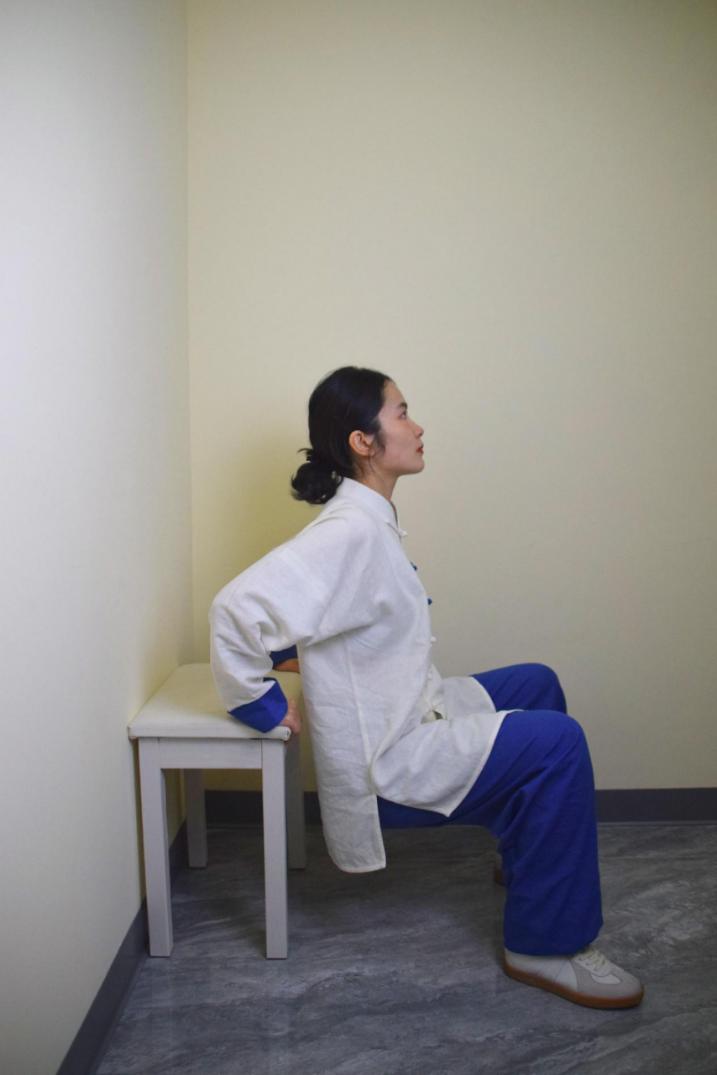
**

**Figure S4. Reverse triceps push-up**

**
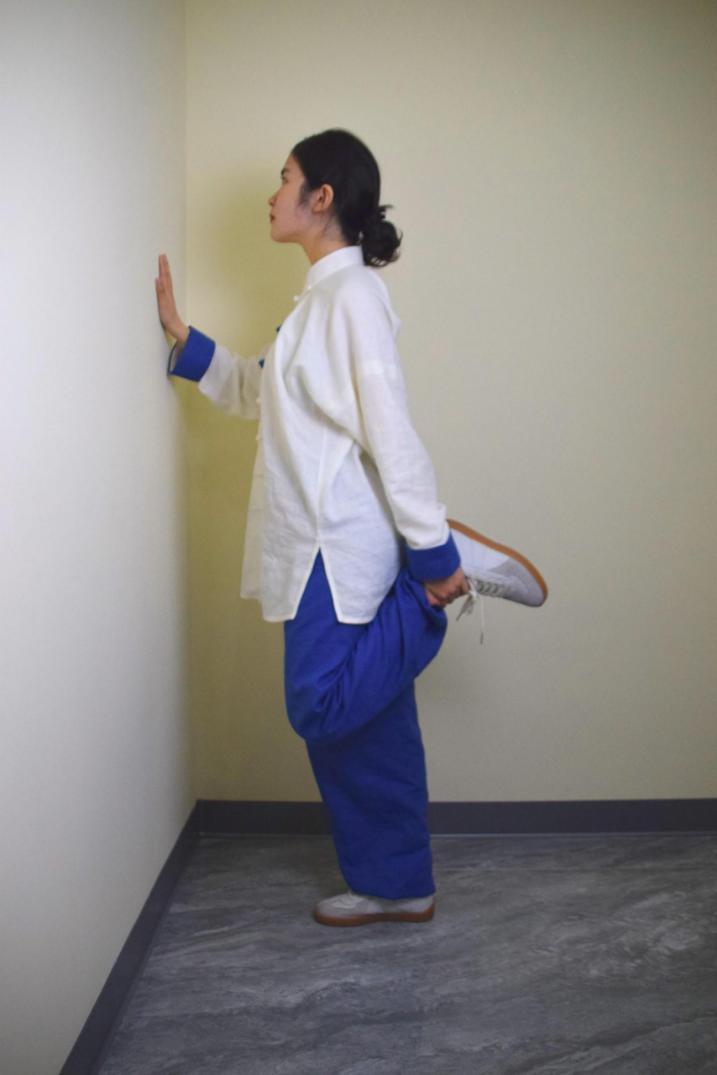
**

**Figure S5. Wall-supported calf raise**


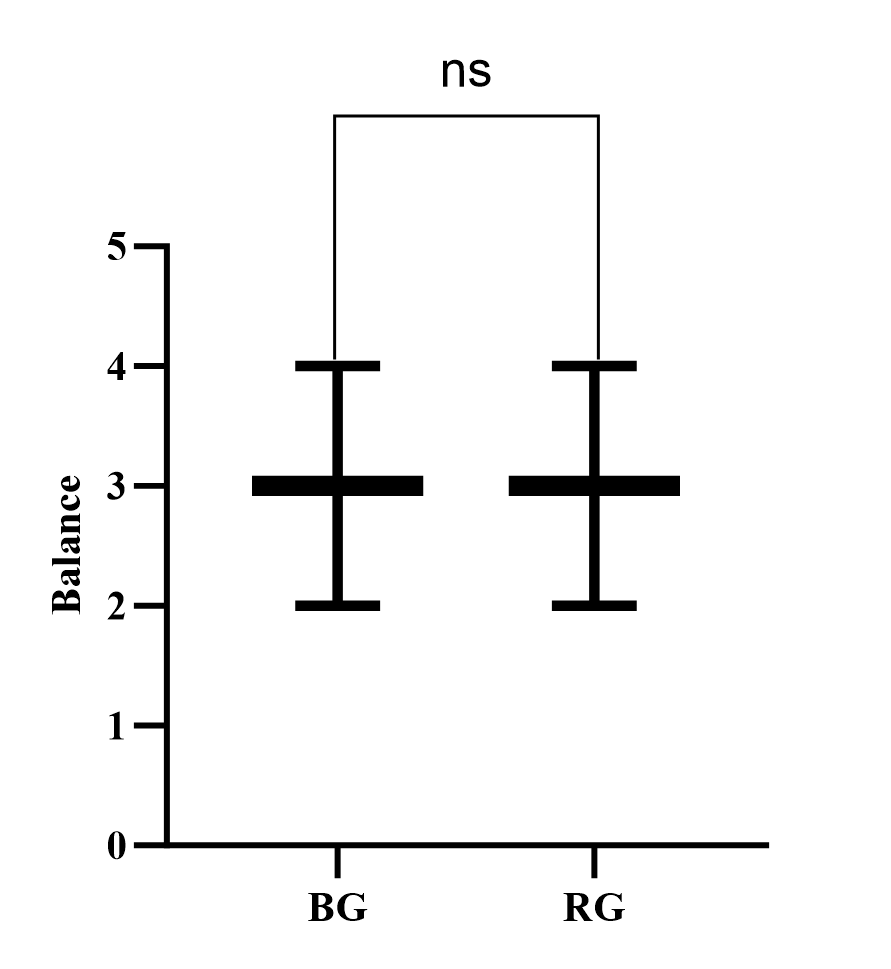


**Figure S6. Comparison of balance between the Baduanjin group and the resistance training group at the 12-week mark of the intervention.**


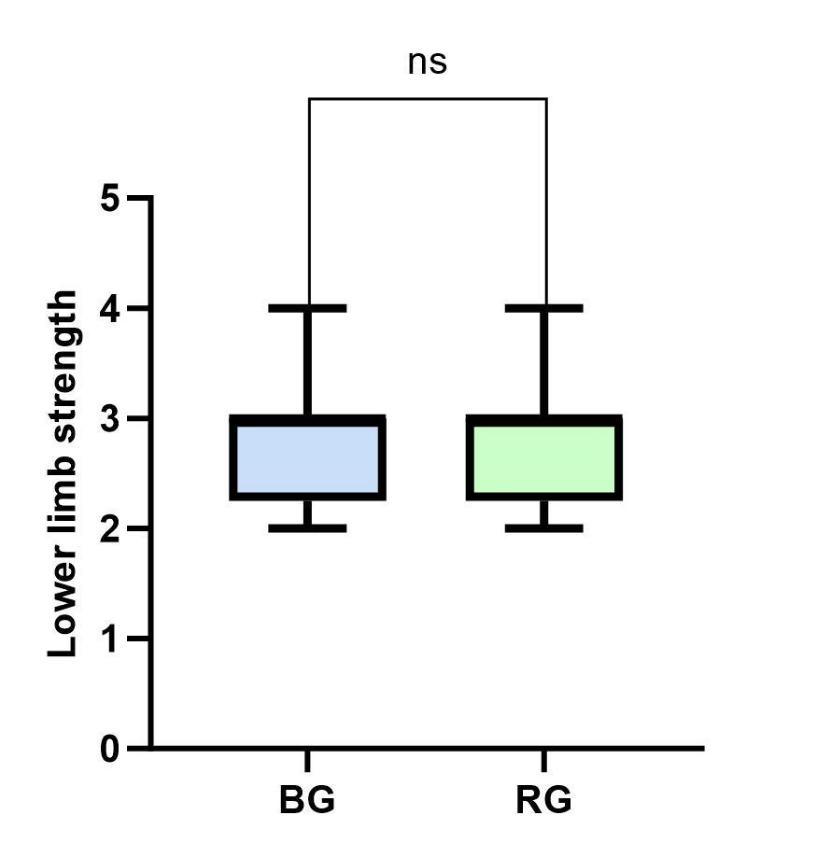


**Figure S7. Comparison of lower limb strength scores between the Baduanjin group and the resistance training group at the 12-week mark of the intervention.**


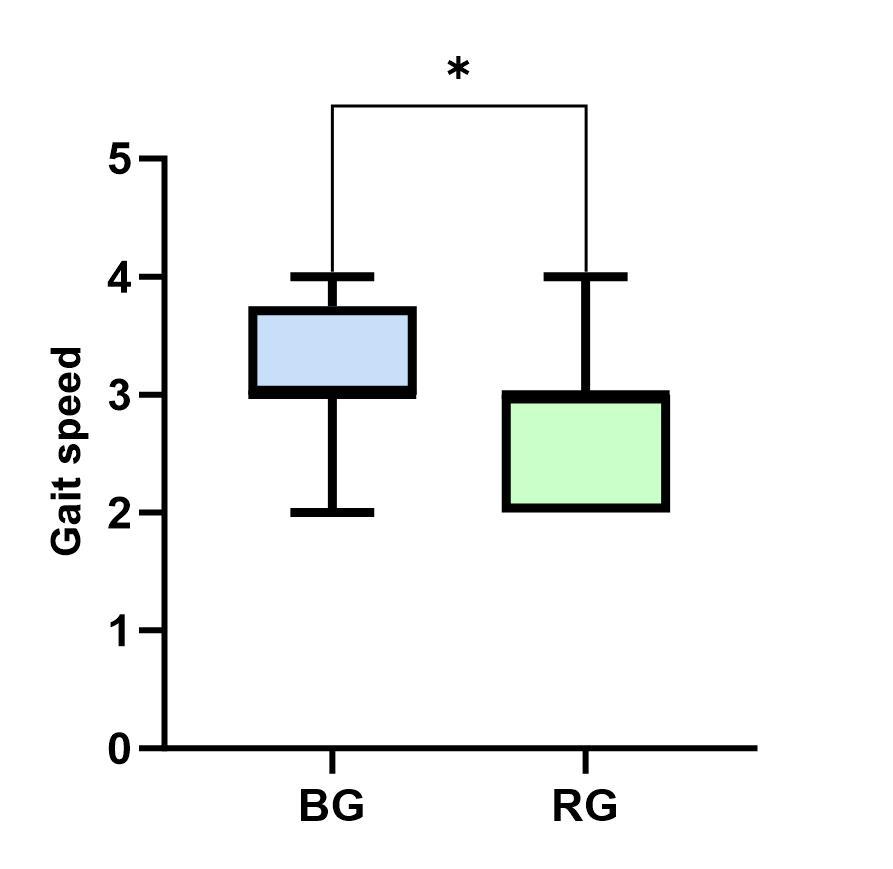


**Figure S8. Comparison of gait speed scores between the Baduanjin group and the resistance training group at the 12-week mark of the intervention.**


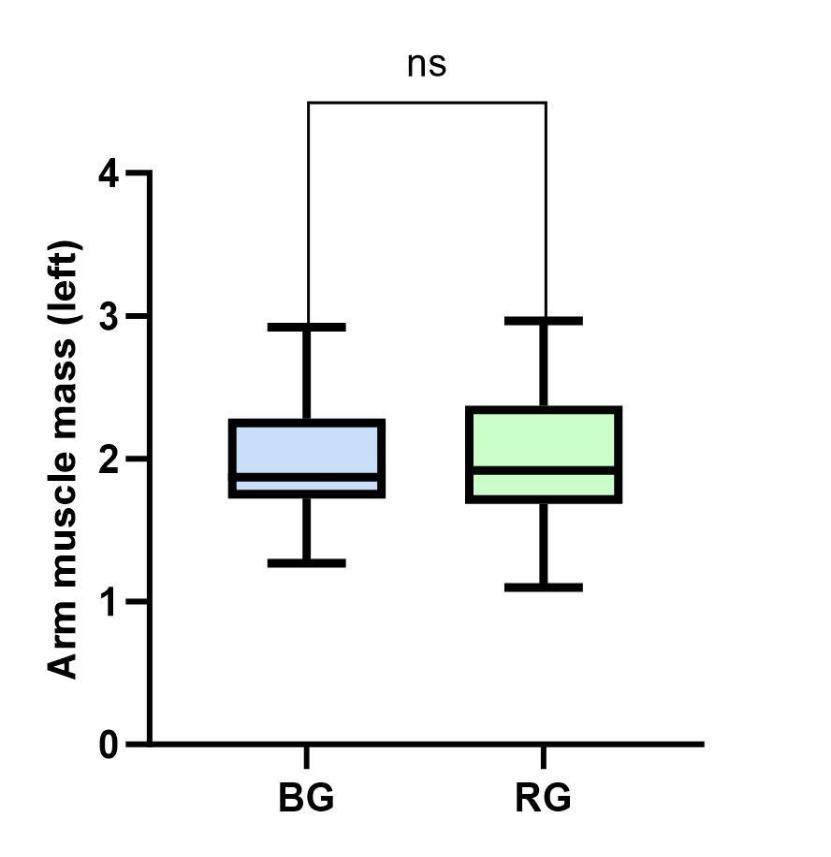


**Figure S9. Comparison of arm muscle mass (left) between the Baduanjin group and the resistance training group at the 12-week mark of the intervention.**


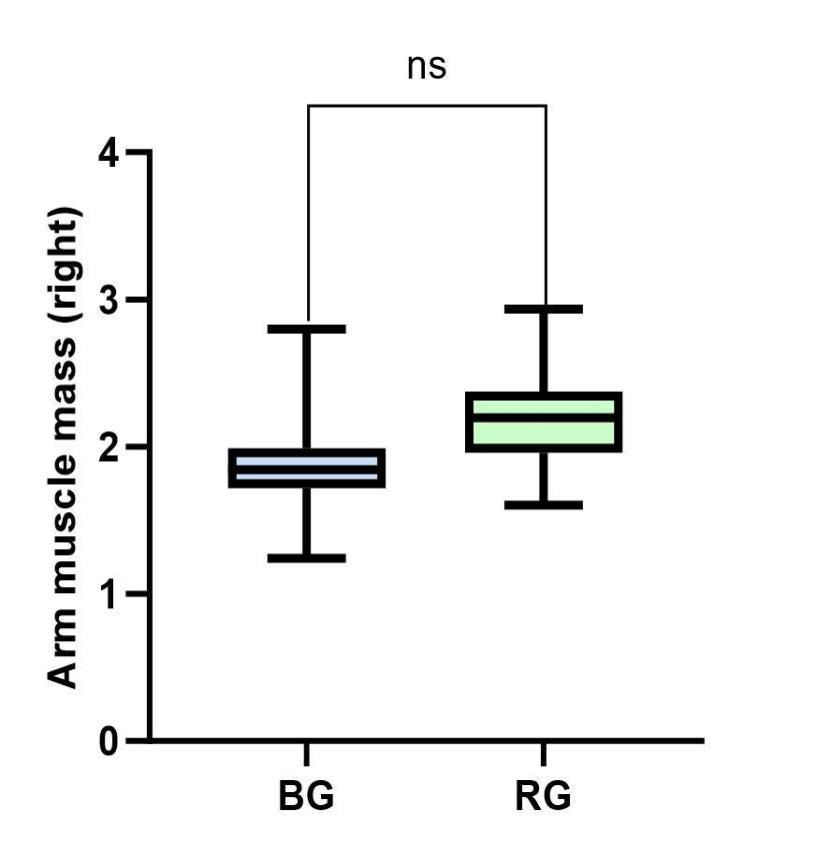


**Figure S10. Comparison of arm muscle mass (right) between the Baduanjin group and the resistance training group at the 12-week mark of the intervention.**


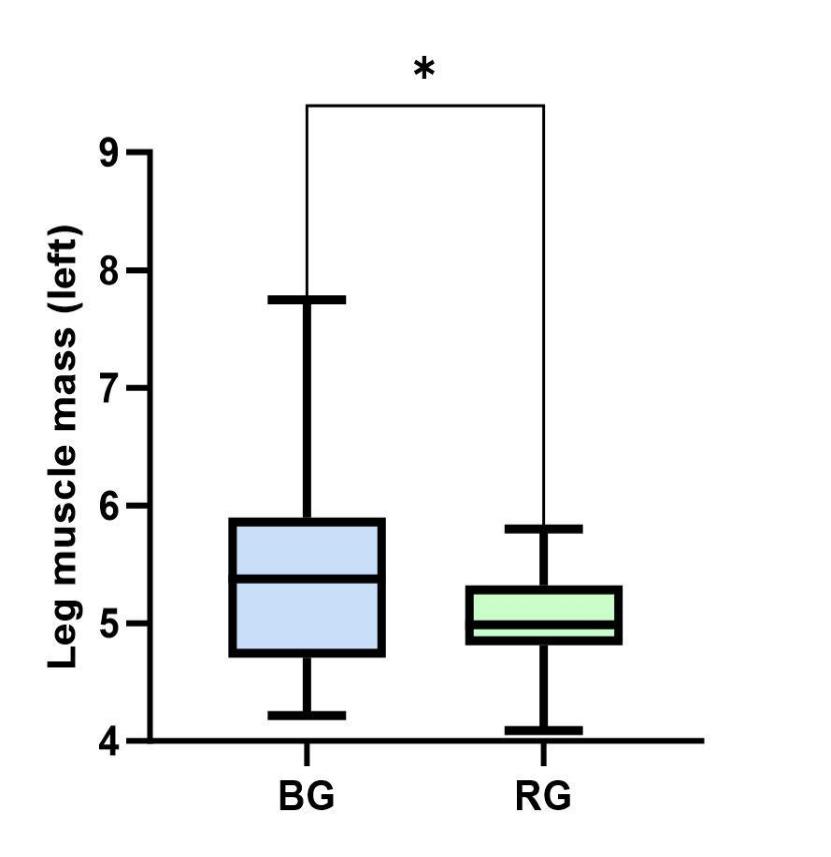


**Figure S11. Comparison of leg muscle mass (left) between the Baduanjin group and the resistance training group at the 12-week mark of the intervention.**


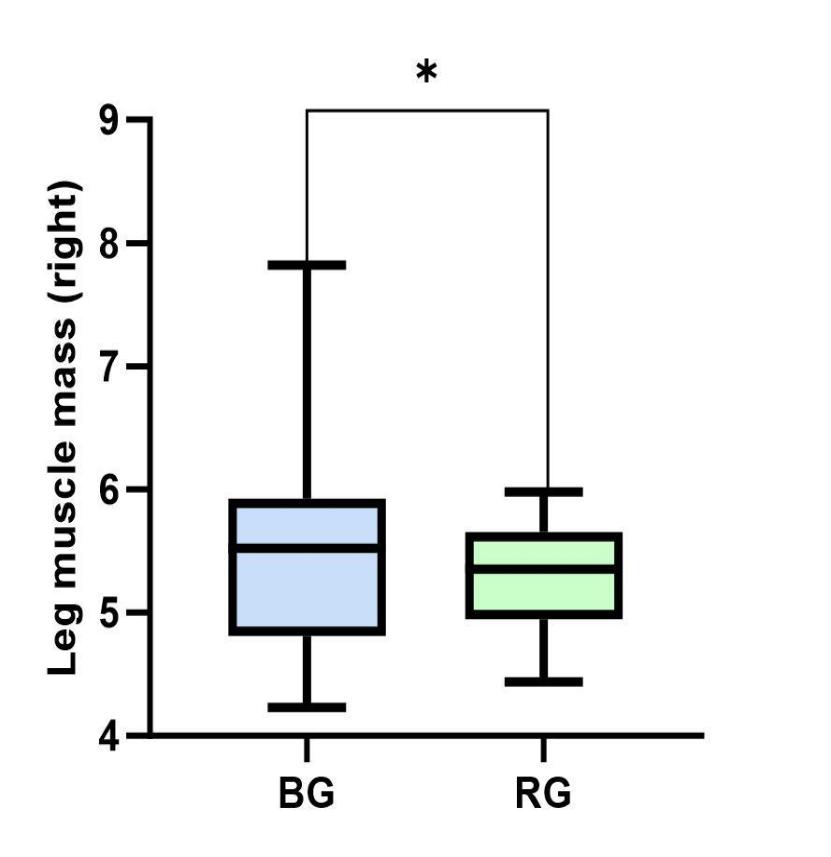


**Figure S12. Comparison of leg muscle mass (right) between the Baduanjin group and the resistance training group at the 12-week mark of the intervention.**


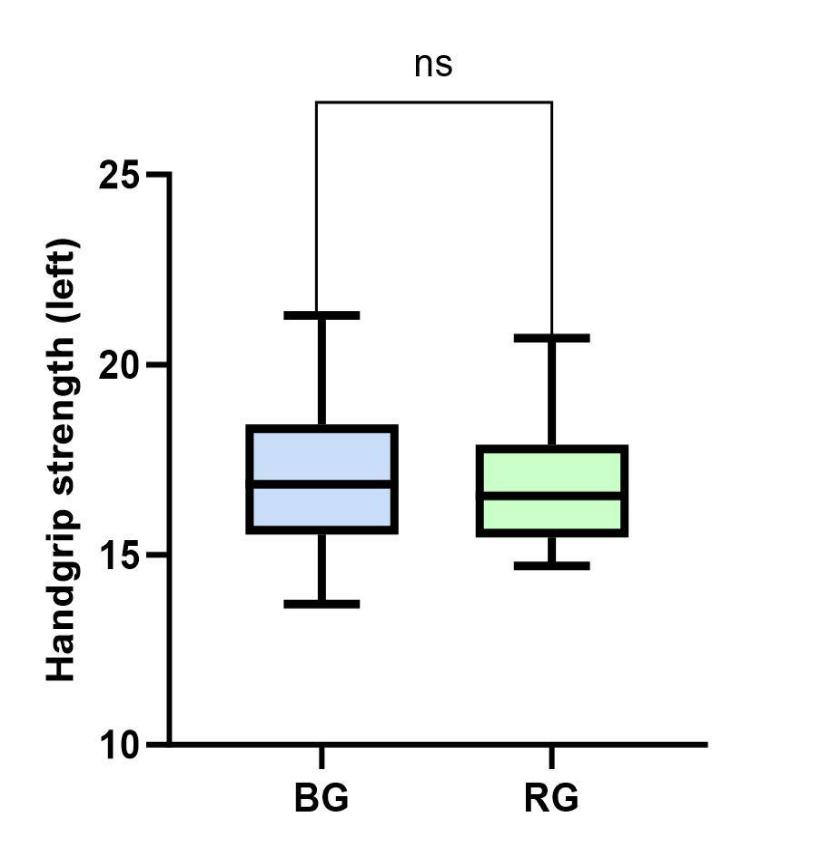


**Figure S13. Comparison of handgrip strength (left) scores between the Baduanjin group and the resistance training group at the 12-week mark of the intervention.**


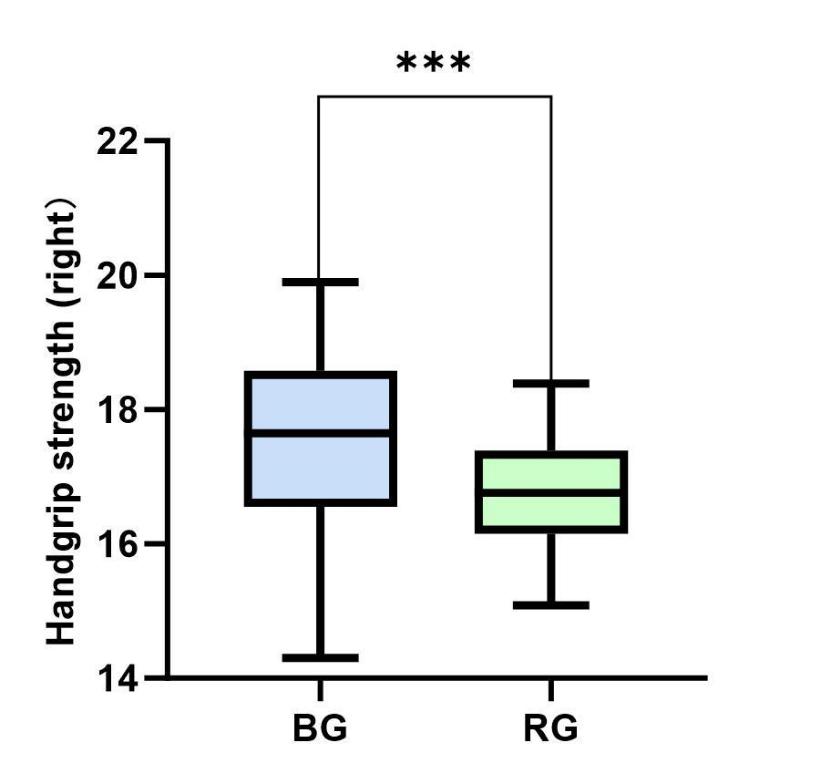


**Figure S14. Comparison of handgrip strength (right) between the Baduanjin group and the resistance training group at the 12-week mark of the intervention.**


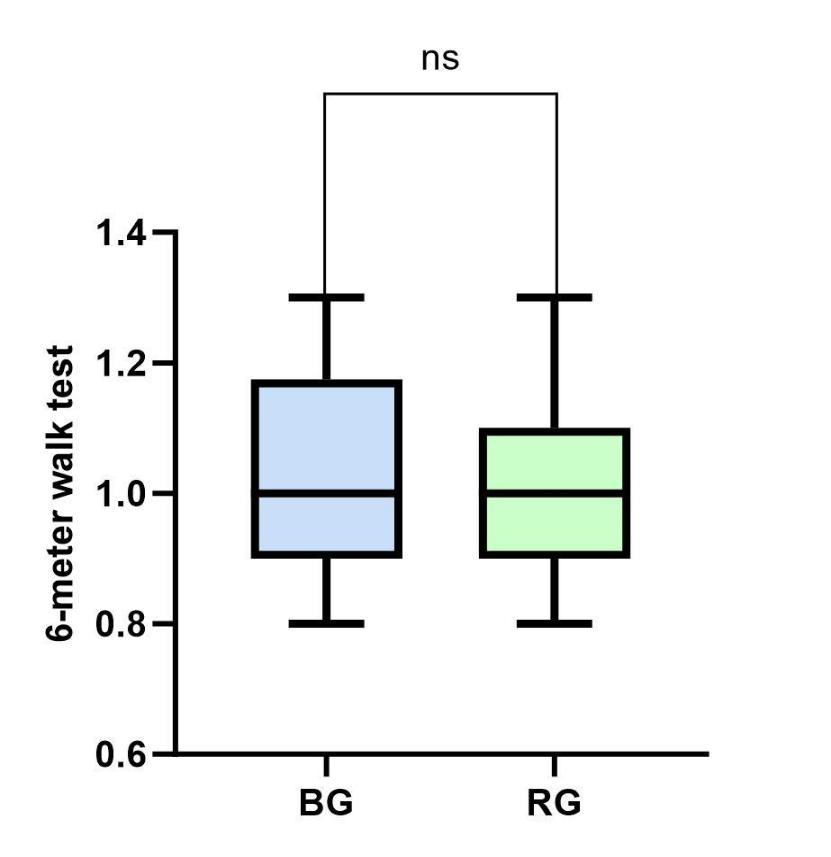


**Figure S15. Comparison of 6-meter walk test between the Baduanjin group and the resistance training group at the 12-week mark of the intervention.**


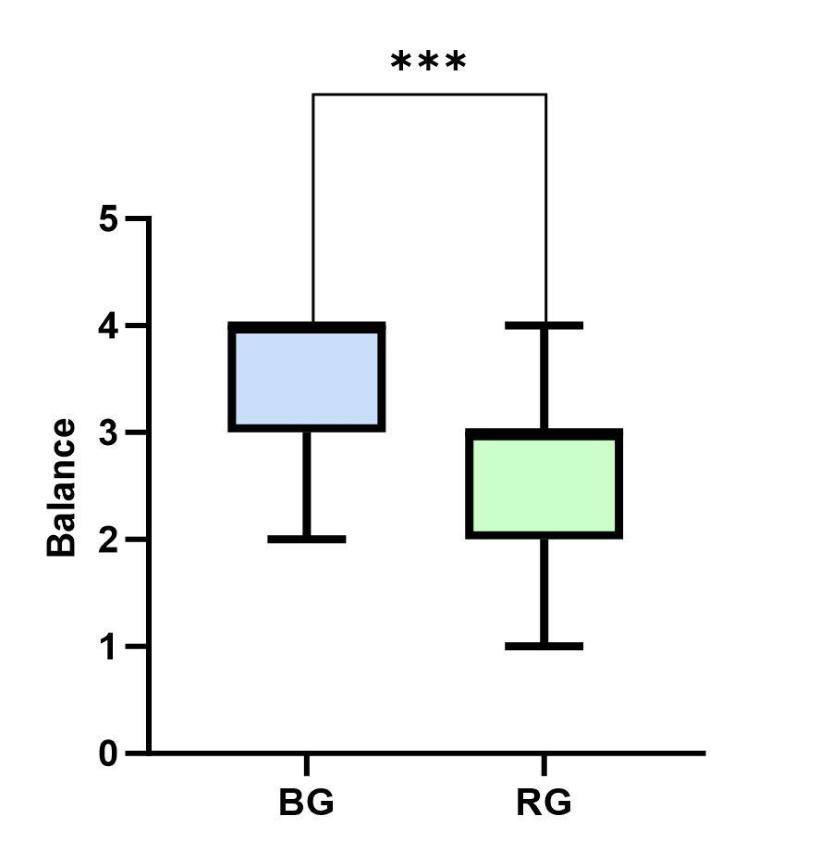


**Figure S16. Comparison of balance between the Baduanjin group and the resistance training group at the 24-week mark of the intervention.**


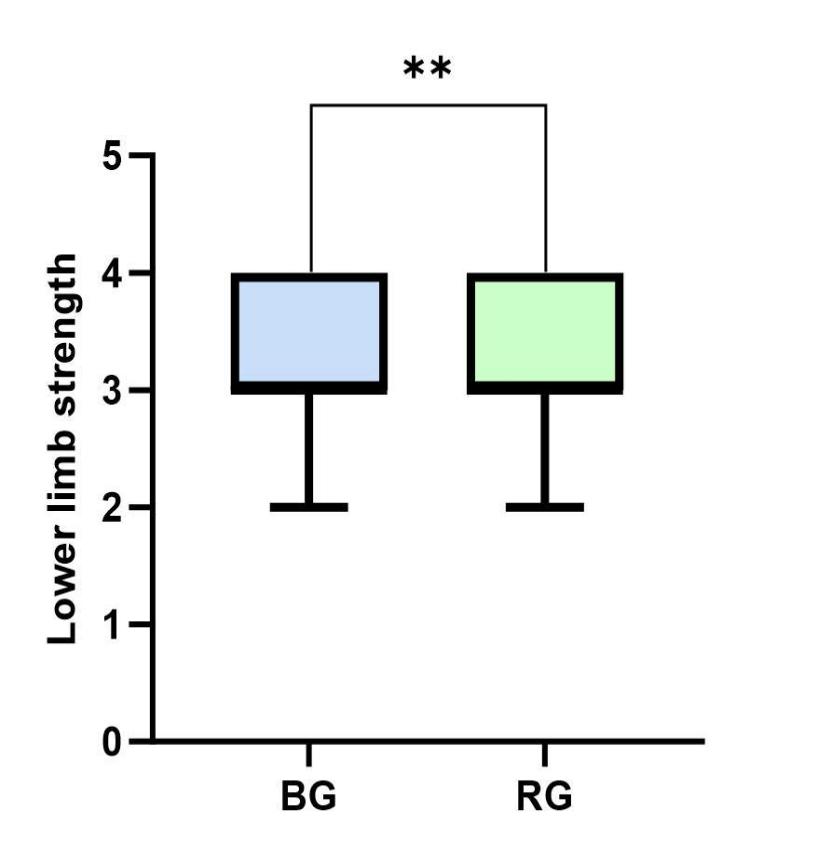


**Figure S17. Comparison of lower limb strength between the Baduanjin group and the resistance training group at the 24-week mark of the intervention.**


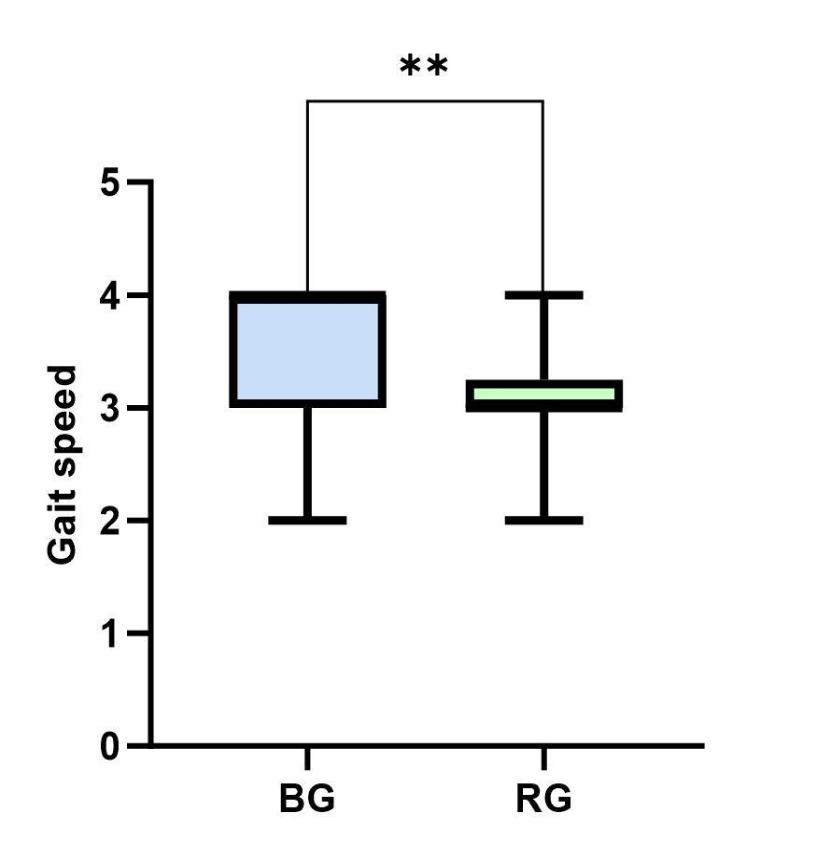


**Figure S18. Comparison of gait speed between the Baduanjin group and the resistance training group at the 24-week mark of the intervention.**


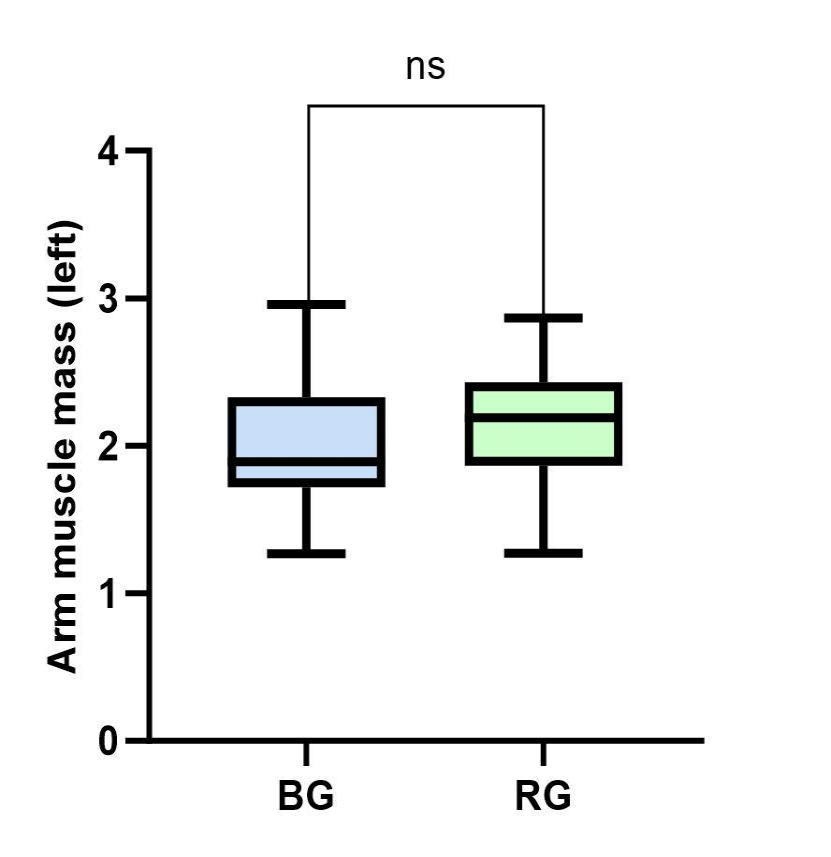


**Figure S19. Comparison of arm muscle mass (left) between the Baduanjin group and the resistance training group at the 24-week mark of the intervention.**


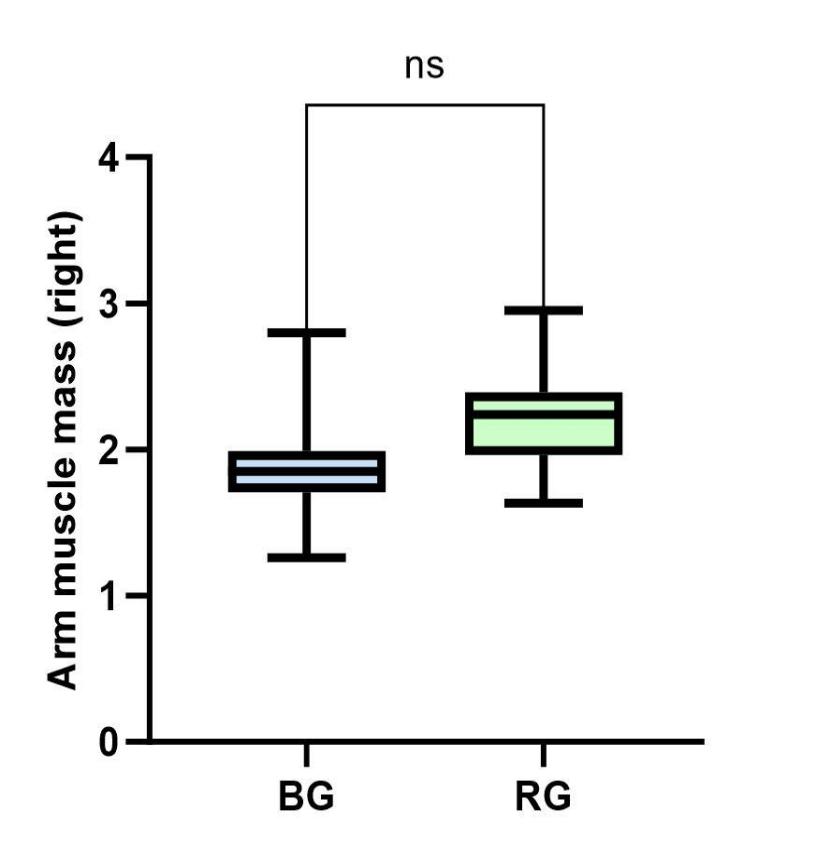


**Figure S20. Comparison of arm muscle mass (right) between the Baduanjin group and the resistance training group at the 24-week mark of the intervention.**


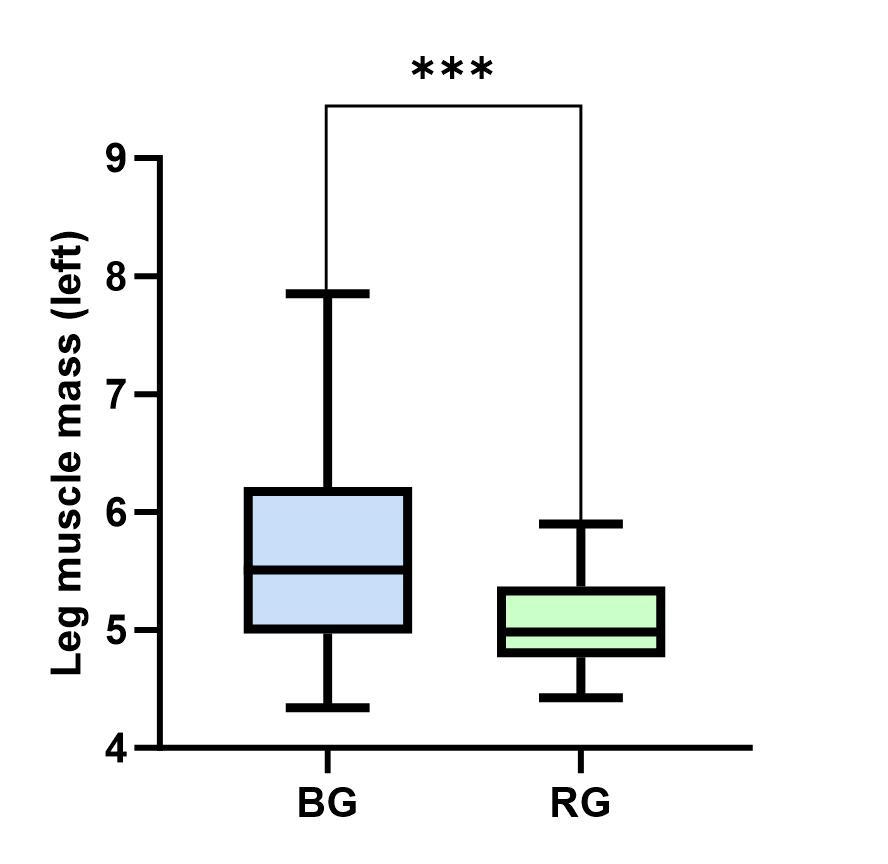


**Figure S21. Comparison of leg muscle mass (left) between the Baduanjin group and the resistance training group at the 24-week mark of the intervention.**


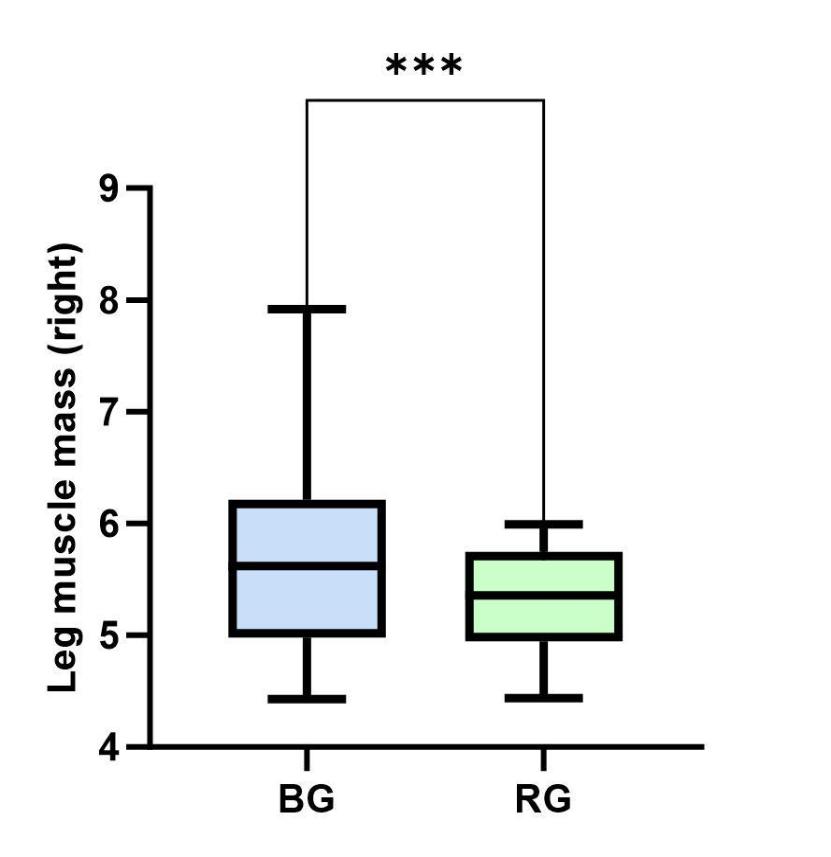


**Figure S22. Comparison of leg muscle mass (right) between the Baduanjin group and the resistance training group at the 24-week mark of the intervention.**


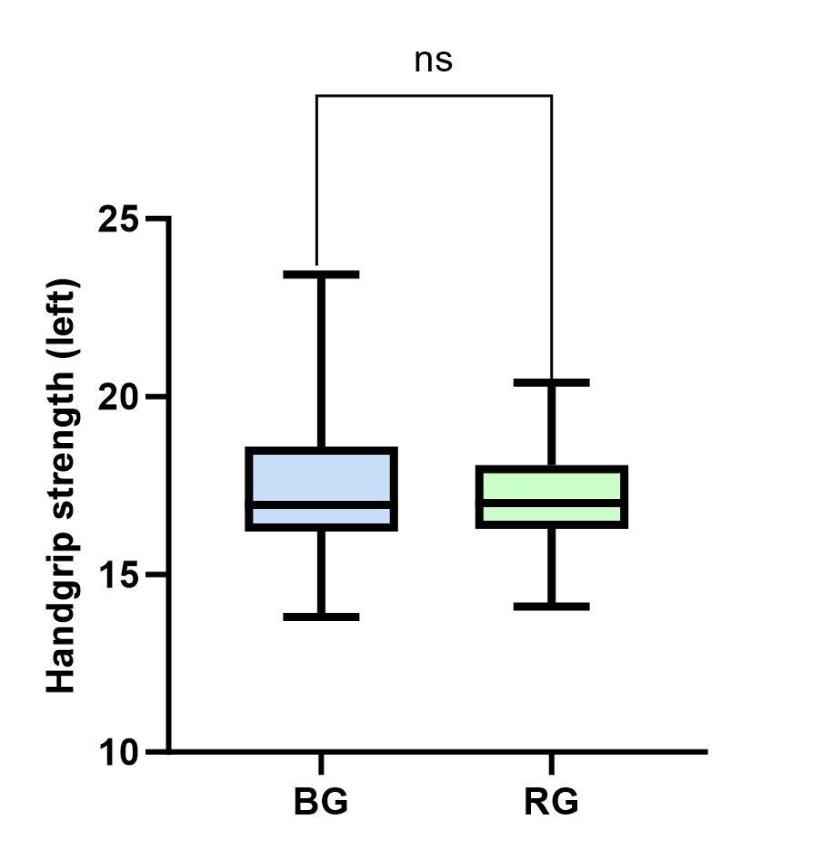


**Figure S23. Comparison of handgrip strength (left) between the Baduanjin group and the resistance training group at the 24-week mark of the intervention.**


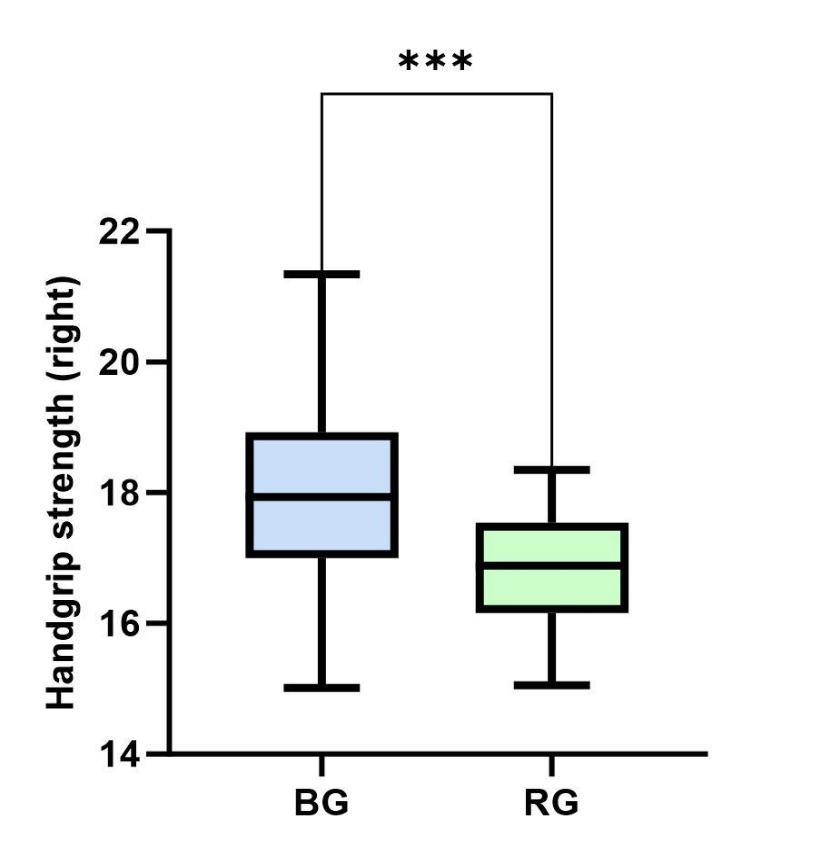


**Figure S24. Comparison of handgrip strength (right) between the Baduanjin group and the resistance training group at the 24-week mark of the intervention.**


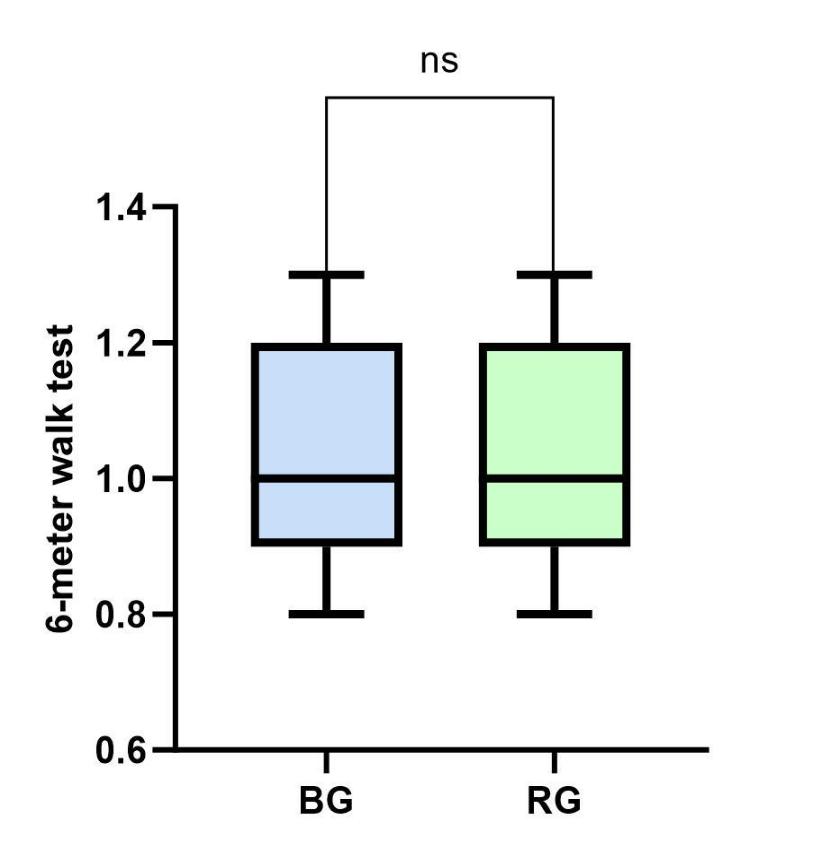


**Figure S25. Comparison of 6-meter walk test between the Baduanjin group and the resistance training group at the 24-week mark of the intervention.**


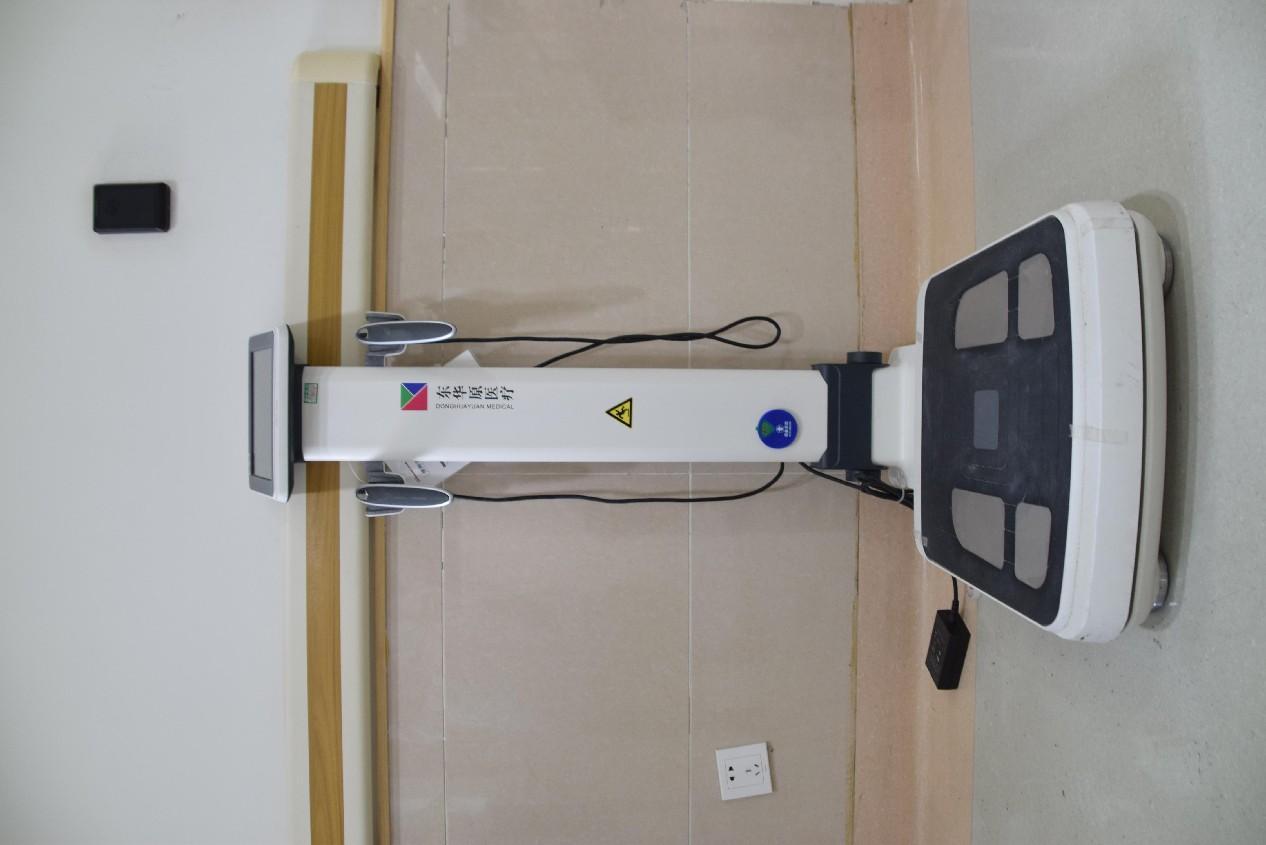


**Figure S26. Donghuahuan body composition analyzer, model DBA-510**

**
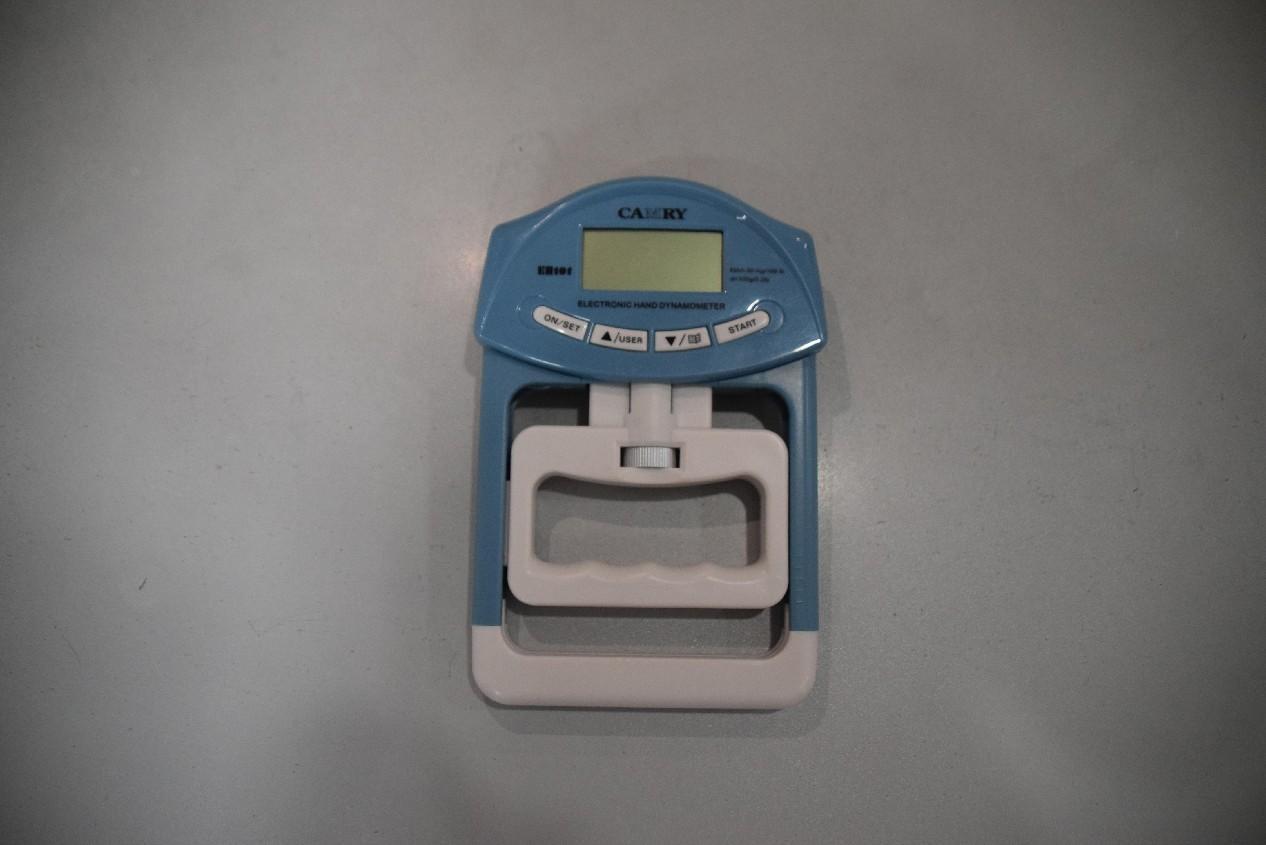
**

**Figure S27. JAMAR grip strength meter**
