# Supplementary material for: Efficacy of Baduanjin Exercise for Sarcopenia in Older Adults: A 24‐Week Randomized Controlled Trial
Source: J Cachexia Sarcopenia Muscle. 2025 Dec 10;16(6):e70163. doi: 10.1002/jcsm.70163 (PMC12695133; doi:10.1002/jcsm.70163)
Supplement: Supplementary file 3 — Data S3: Supporting Information. [file JCSM-16-e70163-s003.docx]

**Data Sharing Statement**

**Data**

**Data available**: Yes

**Data types**: Deidentified participant data

**How to access data**: How to access data: Send request email to Professor Li,

[libin@cdutcm.edu.cn](mailto:libin@cdutcm.edu.cn)

**When available**: With publication

**Supporting Documents**

**Document types**: None

**Additional Information**

**Who can access the data**: anyone requesting the data

**Types of analyses**: for any purpose

**Mechanisms of data availability**: with investigator support
